# Survival and growth of C57BL/6J mice lacking the BK channel, *Kcnma1*: lower adult body weight occurs together with higher body fat

**DOI:** 10.14814/phy2.13137

**Published:** 2017-02-27

**Authors:** Susan T. Halm, Michael A. Bottomley, Mohammed M. Almutairi, Maurico Di Fulvio, Dan R. Halm

**Affiliations:** ^1^ Department of NeuroscienceCell Biology and PhysiologyWright State University Boonshoft School of MedicineDaytonOhio; ^2^ Department of Mathematics and StatisticsStatistical Consulting CenterWright State UniversityDaytonOhio; ^3^ Department of Pharmacology and ToxicologyWright State University Boonshoft School of MedicineDaytonOhio

**Keywords:** Body composition, breeding success, environmental stress, glucose tolerance, mouse development, quantitative magnetic resonance

## Abstract

Big conductance potassium (BK) channels contribute to K^+^ flow and electrical behavior in many cell types. Mice made null for the gene (*Kcnma1*) producing the BK channel (BK^KO^) exhibit numerous deficits in physiological functions. Breeding mice lacking a single allele of *Kcnma1* (C57BL/6J background) had litter sizes of approximately eight pups. For the period of maternal care (P0–P21), pup deaths peaked at P1 with a second less severe interval of death peaking near P13. Early deaths were twice as likely during a 20‐month period of building construction compared with the quiescent period after cessation of construction. Births during construction were not consistent with Mendelian predictions indicating the likelihood of a specific disadvantage induced by this environmental stressor. Later BK^KO^ pup deaths (~P13) also were more numerous than Mendelian expectations. After weaning, weight gain was slower for BK^KO^ mice compared with wild‐type littermates: 5 g less for male BK^KO^ mice and 4 g less for female BK^KO^ mice. Body composition determined by quantitative magnetic resonance indicated a higher fat proportion for wild‐type female mice compared with males, as well as a higher hydration ratio. Both male and female BK^KO^ mice showed higher fat proportions than wild‐type, with female BK^KO^ mice exhibiting greater variation. Together, these results indicate that BK^KO^ mice suffered disadvantages that lead to prenatal and perinatal death. A metabolic difference likely related to glucose handling led to the smaller body size and distinct composition for BK^KO^ mice, suggesting a diversion of energy supplies from growth to fat storage.

## Introduction

Potassium channels contribute to the electrical properties of membranes, revealing the orientation and magnitude of K^+^ concentration gradients. The typically high intracellular and low extracellular K^+^ concentrations lead to cell membrane electrical potential differences (*V*_m_) negative on the inside compared with the outside. Instead of using a single gene to produce K^+^ channel proteins, the mammalian genome contains a family of over 70 K^+^ channel genes (González et al. 2012). From this multitude of largely similar proteins, a wide range of subtly distinctive regulatory controls allow cells to increase or decrease membrane K^+^ conductance and thereby adjust the magnitude of *V*_m_. The Big conductance potassium (BK) channel (*Kcnma1*, K_Ca_1.1, maxi‐K, *slo1*) is a large conductance, voltage‐regulated, and Ca^2+^‐regulated K^+^ channel from this protein family found in numerous cell types (Contreras et al. 2013). In many cells, BK activity suppresses action potential excitability and/or vesicular release of signaling molecules. For certain epithelial cells, the BK channel serves as a primary route for K^+^ exit across the apical membrane that completes the transepithelial flow necessary for lowering body K^+^ levels as performed in the kidney or for raising luminal K^+^ concentration as found in the colon (Wen et al. 2014; Carrisoza‐Gaytan et al. 2016; Halm 2016). Together, the myriad expressions of the BK channel make it a crucial membrane component allowing optimal cellular performance.

Production of mice lacking expression of the BK channel (global knockout, BK^KO^) provided a model to demonstrate the influences of this K^+^ channel type at the level of a complete organism (Meredith et al. 2004; Sausbier et al. 2004). Overall these mice exhibit many deficits, but none of the attendant issues rise to the level that precludes survival by a reasonable cohort of BK^KO^ mice. Although both female and male BK^KO^ mice are fertile, small litter size makes breeding of heterozygotes the most expedient means to generate BK^KO^ mice for study (Meredith et al. 2004). Several prominent deficits include ataxia, weak grip, hearing loss, circadian imbalances, and urinary bladder incontinence (Meredith et al. 2004, 2006; Rüttiger et al. 2004; Sausbier et al. 2004).

Inbred mouse strains often carry spontaneously arising silent mutations that could influence the phenotype observed for mice made to lack a specific gene product (Petkov et al. 2004; Taft et al. 2006; Stevens et al. 2007; Mekada et al. 2009; Zurita et al. 2011). The resulting interactions might magnify the difficulty encountered or mask a deficit until a stressor is applied to a mouse. For this reason, the specific background strain of the mice lacking BK likely alters the severity of the physiological outcomes. In particular, the C57BL/6J mouse strain exhibits blunted glucose‐stimulated insulin secretion from isolated pancreatic islets (Berglund et al. 2008; Fergusson et al. 2014), possibly a consequence of lacking nicotinamide nucleotide transhydrogenase (Freeman et al. 2006; Wong et al. 2010; Ronchi et al. 2013). Additionally, mice of different genetic backgrounds exhibit distinct patterns of insulin secretion, with FVB/N mice exhibiting hepatic insulin resistance and marginal insulin secretion in response to a glucose load, but high responsiveness to insulin‐induced hypoglycemia (Berglund et al. 2008).

Results in this report provide insights into the survival of BK^KO^ mice during development as well as their growth and body composition. Because the BK channel often serves to adjust ongoing activity, the genome of the background mouse strain (C57BL/6J in this study; Mekada et al. 2009; Zurita et al. 2011) imposes a key set of biases onto the adaptability conferred by the BK channel. Unintended stresses in the animal vivarium also can skew results or contribute additional experimental variables requiring appropriate controls (Dallman et al. 1999). During this study, construction of a building adjacent to the vivarium influenced pup survival. This unfortunate calamity turned to opportunity, in particular, by using records of breeding success and weight gain that allowed this influence to be quantified. Overall, the impact of lacking a specific protein likely extends broadly, requiring attention to subtle variations to demonstrate the whole‐body physiologic state of these modified animals.

## Material and Methods

Mouse breeding was conducted in the Laboratory Animal Resources facility of Wright State University (LAR) as described in an Animal Use Protocol issued by the WSU Institutional Animal Care and Use Committee. BK null mice were produced by breeding mice lacking a single allele (+/−) of the *Kcnma1* (slo1) gene. The initial breeding pairs in a C57BL/6J background strain were obtained from Dr. Andrea Meredith at the University of Maryland (http://meredithlab.org/). During this study, the WSU colony reached 8 generations and was not backcrossed with C57BL/6J mice, because backcrossing to maintain the background strain is recommended at 10 generations. The mice received standard chow (Teklad 22/5 Rodent Diet 8640) and water ad libitum, and were housed in cages (Mouse Cage PCT2L12SHT, 8.4″ W × 14.25″ L × 5.2″ H in PNC Racks; Allentown, Inc., Allentown, NJ) with same sex littermates. Breeding pairs were housed together until signs of pregnancy were noted, at which time the male was removed. Pups were tail clipped for genotyping at an age of 5–8 days and designated: WT, wild‐type; het, heterozygotic; KO, BK knockout (null). Weaning occurred at an age of ~21 days for WT and het pups, with the KO pups generally kept with the dams until an age of ~28 days to improve survival. Weaned KO pups were provided moistened chow in a container on the bottom of the cage until an age of 6 weeks to assure weight gain. Anecdotally, WT and het mice often ate while fully inverted by grasping onto the wire cage top. KO mice rarely maintained grip on the cage top long enough to approach the feeding tray in this manner.

### Genotyping

Genomic DNA was extracted by DNeasy tissue kit (Qiagen, Valencia, CA) from isolated tail segments. Briefly, DNA was amplified by PCR with primers to detect WT and KO segments: initial denaturing 94°C (2 min), 5 cycles of denaturation 94°C (30 sec), annealing 55°C (30 sec), extension 68°C (2 min), 30 cycles of denaturation 94°C (30 sec), annealing 50°C (30 sec), extension 68°C (2 min), final extension 72°C (5 min). Primers for *Kcnma1* exon1 segment were *forward* 5′‐TTC‐ATC‐ATC‐TTG‐CTC‐TGG‐CGG‐ACG‐3′ with *reverse* 5′‐CCA‐TAG‐TCA‐CCA‐ATA‐GCC‐C‐3′. Primers for KO Neo segment were *forward* 5′‐ATA‐GCC‐TGA‐AGA‐ACG‐AGA‐TCA‐GC‐3′ with *reverse* 5′‐CCT‐CAA‐GAA‐GGG‐GAC‐TCT‐AAA‐C‐3′.

### General glucose metabolism

Evaluation of glucose homeostasis followed the recommendations of the International Mouse Phenotyping Resource of Standardized Screens (Ayala et al. 2010). Intraperitoneal (ip) glucose and insulin tolerance tests (ipGTT and ipITT) were performed by ip administration of 2 g/kg d‐glucose or 0.75 U/kg insulin, respectively, to conscious mice. Mice were fasted overnight prior to these tests to assure depletion of glycogen stores. Blood glucose was determined using a calibrated glucometer (FreeStyle‐Lite, Abbott, Abbott Park, IL).

### Body composition

Mice were weighed (CS200 balance, Ohaus, Parsippany, NJ) weekly after weaning to monitor growth and overall health, generally at midweek. Measurement of food intake was discontinued because of variability associated with recovery of uneaten pellet crumbs from the bedding material (Tobin et al. 2007). Body composition of colony mice was determined by quantitative magnetic resonance (QMR) on the same day as the weekly weighing using an EchoMRI Analyzer (EchoMRI LLC, Houston, TX; http://www.echomri.com/) that provided measures of lean mass, fat mass, total water mass, and free water mass (Tinsley et al. 2004; Kovner et al. 2010; Nixon et al. 2010). After calibration with a canola oil standard, mice were restrained in a plexiglass tube for 60–90 sec during composition measurements, and then returned to their housing cages. Fat mass records all fat molecules in the body expressed as an equivalent mass of canola oil. Lean mass records the muscle tissue mass equivalent of all body parts containing water, which excluded fat, bone minerals, and substances not contributing to the NMR signal, such as hair and claws. Contributions to free water represent mostly bladder contents. Total water includes both the free water and the water contained in the lean component with a hydration ratio ([Total Water − Free Water]/Lean) of ~80% for control animals. Total analyzer weight consists of lean, fat, free water, undetectable substances, and systematic errors, such that the calculated total (Total = Lean + Fat + Free Water) estimates total body weight.

### Data analysis

Records of pup genotypes and sex were compared with expectations of binomial distributions from the Mendelian segregation of alleles using chi‐square (*χ* ^2^) tests of the null hypothesis (Montoliu 2012; Papaioannou and Behringer 2012). Mendelian expectations were calculated using the total number of mice in the group. Categories generally included six genders: mWT, male wild‐type; fWT, female wild‐type; mhet, male heterozygote; fhet, female heterozygote; mKO, male knockout; and fKO, female knockout. For smaller samples, sexes were combined resulting in three categories: WT, het, and KO. Those categories having a chi‐square larger than the *P* = 0.05 value for one degree of freedom (3.84) were noted as major contributors to falsifying null hypotheses. The Mendelian character of births was examined further by calculating a ratio of observed/expected for gender categories in each litter to provide a measure of variation in these relative deviations from binomial expectations.

Another Mendelian model was calculated motivated by the likelihood that some pups were lost from categorization due to prenatal death. The standard Mendelian model assumes that all subjects in the group were categorized. Assuming instead that mWT and mhet pups were not specifically advantaged or disadvantaged, a second Mendelian prediction (*Mendelian2*) can be obtained from this alternative number of total pups, *n* ^*t*^_p_ = 8/3 (*n*_mwt_ + *n*_mhet_). Minimizing the sum of the chi‐square components for the mWT and mhet categories refined *n* ^t^_p_ further. This second model only accounts for losses that occur by non‐Mendelian mechanisms; any losses occurring through a mechanism involving Mendelian proportions still will be missed.

Histograms of mouse weights in each gender category were analyzed with an average shifted histogram procedure to provide estimates of category weight distributions (Scott 2015). Binning and smoothing parameters were chosen to maintain a similar resolution for the categories within each comparison. Mean body composition values for each gender category were obtained as the mean of the mice measured; the value for each mouse was determined from the mean of weekly measurements (2–4).

Results are reported as mean and standard deviation (SD) or standard error of the mean (SEM) with the number of animals (*n*) or litters (*N*) indicated. Statistical comparisons were made using two‐tailed Student's *t*‐test, with significant difference accepted provisionally at *P* ≤ 0.05. Bonferroni corrections were made to control for potentially inflated Type I error due to multiple measures. Statistical differences among the gender categories were assessed by littermate comparisons (Zorrilla 1997). The body composition dataset was fit to a mixed analysis of variance (ANOVA) model for gender and litter effects including a stepdown Bonferroni multiple comparison procedure to obtain adjusted *P* values (Holm 1979; Bender and Lange 2001; Gordi and Khamis 2004). All ANOVA was performed using SAS version 9.4.

## Results

### Pup survival – postnatal

At weaning, the number of both mKO and fKO mice were fewer than anticipated based on the number of mWT and fWT littermates. A comparison of the weanling progeny to a binomial distribution indicated a failure of the null hypothesis (*P* < 0.0001, Table 1); the six gender categories did not follow Mendelian expectations for heterozygous mating. In addition, the male proportion of weaned pups (52.7%, *χ* ^2^ = 1.85, *P* = 0.17) conformed to expectations of 50%. Seemingly, decreased survival of KO pups was independent of sex. This deficit of KO pups supported the onset of a developmental requirement for the BK channel prior to weaning.

**Table 1:** Genotype and sex of pups weaned

	mWT	fWT	mhet	fhet	mKO	fKO	*n* ^w^_p_	*N* ^w^_L_
Observed	102	88	168	145	59	62	624	95
*Mendelian*	78	78	156	156	78	78		
Delta	+24	+10	+12	−11	−19	−16		
*χ* ^2^	*7.38*	*1.28*	*0.92*	*0.78*	*4.63*	*3.28*	*18.27*,* P* < 0.0001	
*Mendelian2*	90	90	181	181	90	90	**723**	95
Delta	+12	−2	−13	−36	−31	−28		
*χ* ^2^	*1.49*	*0.06*	*0.90*	*7.07*	*10.89*	*8.91*	*29.3*	

Genotype and sex of pups were determined for each weaned litter ($N_{L}^{W}$). The number of weaned pups were compared with a Mendelian model based on the total number of weaned pups ($n_{p}^{W}$) or the number of mWT and mhet pups weaned (bold, *Mendelian2*, see Methods). Individual categories deviating from these Mendelian models with a chi‐square larger than the value for *P* = 0.05 at one degree of freedom (3.84) are highlighted. The average number of pups born ($n_{p}^{b}$) was 8.5 ± 2.0 pups/litter and the average number weaned ($n_{p}^{W}$) was 6.6 ± 2.5 pups/litter (mean ± SD). Bold value indicates predicted total number based on the logic in methods. Italics values indicate chi square values.

**Figure 1:**
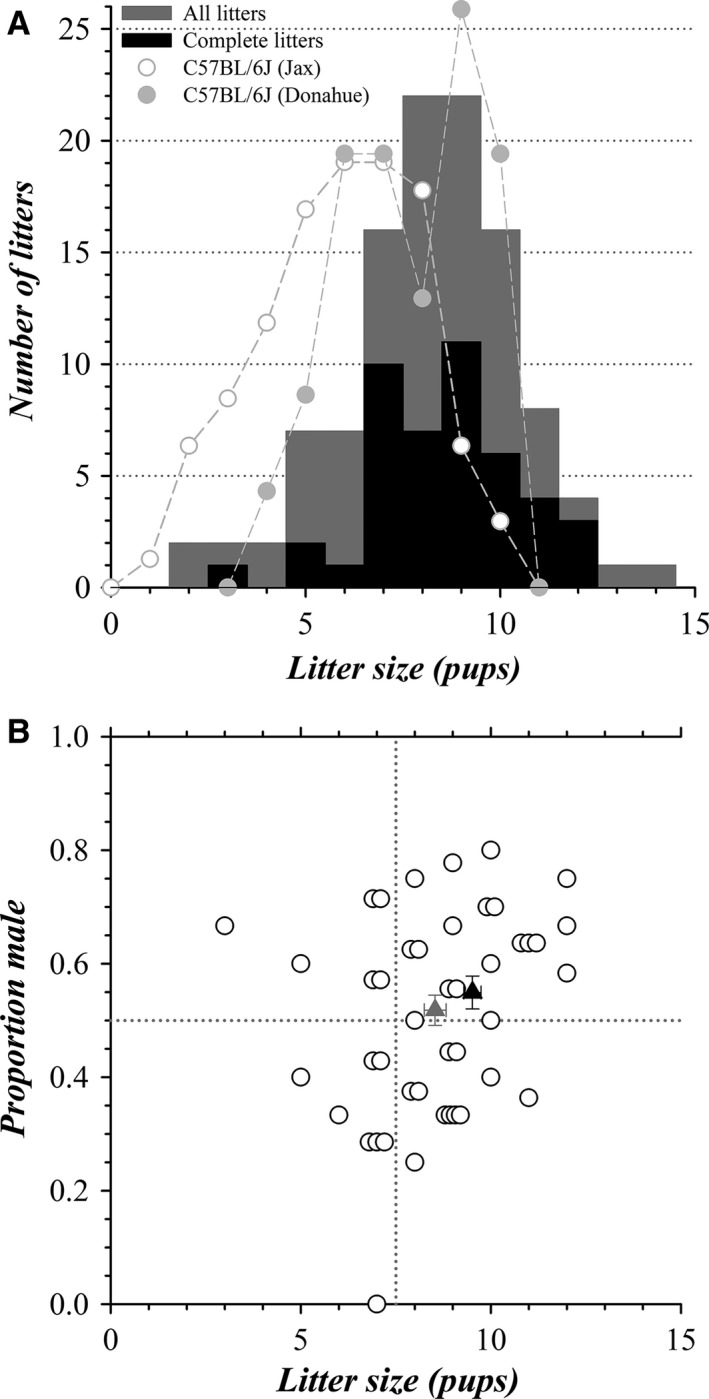
Characteristics of colony births: litter size and sex distribution. (A) The distribution of litter sizes is plotted (gray bars, total litters = 110, total pups = 904), together with the distribution of sizes for complete litters (black bars, complete litters = 45, pups = 384). Litter size distributions also are shown for C57BL/6J breeding colonies normalized to the total number of WSU colony litters (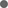, 51 litters, Mouse Phenome Donahue19 dataset, reproduction‐fecundity, number of pups born per litter, C57BL/6J; Donahue 2016; ○, 260 litters, Mouse Phenome Jax5 dataset, reproduction‐fecundity, number of born per litter, C57BL/6J; The Jackson Laboratory 2016). (B) The proportion of males present in each complete litter is plotted (○), together with the mean for all complete litters (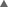, 0.52 ± 0.03 mean ± SEM, litter number = *N*_L_ = 45; different from 0.50, *P* = 0.51) and complete litters with ≥8 pups (▲, 0.55 ± 0.03, *N*_L_ = 31; different from 0.50, *P* = 0.10).

The distribution of litter sizes exhibited a modal value of 8.5 pups/litter, ranging from 2 to 14 pups/litter (gray bars, Fig. 1A). Some pup deaths in the first few days after birth likely went unnoticed, because dams often scavenge dead pups (Weber et al., 2013a), possibly contributing to a leftward shift in the litter size distribution. Because sex and genotype generally were determined only at an age of 5–8 days, the group of completely categorized litters was smaller (black bars, Fig. 1A). Of these complete litters, the proportion of male pups ranged from 0.0 to 0.8, relatively independent of litter size (Fig. 1B). Examining only the litters with ≥8 pups likely reduces bias resulting from any undocumented deaths. The intrauterine position of female fetuses in relation to male fetuses influences the sex ratio of subsequent litters delivered by those female mice as dams (Vandenbergh and Huggett 1994). Female fetuses gestating with two adjacent male fetuses will, as dams, have a higher proportion of male pups in each litter and those gestating with two adjacent female fetuses will have a lower proportion of males. The wide range in proportion of male pups (Fig. 1B) suggests that the dams included a random selection of fhet mice that had gestated in an intrauterine position either with adjacent male fetuses or with adjacent females.

**Figure 2:**
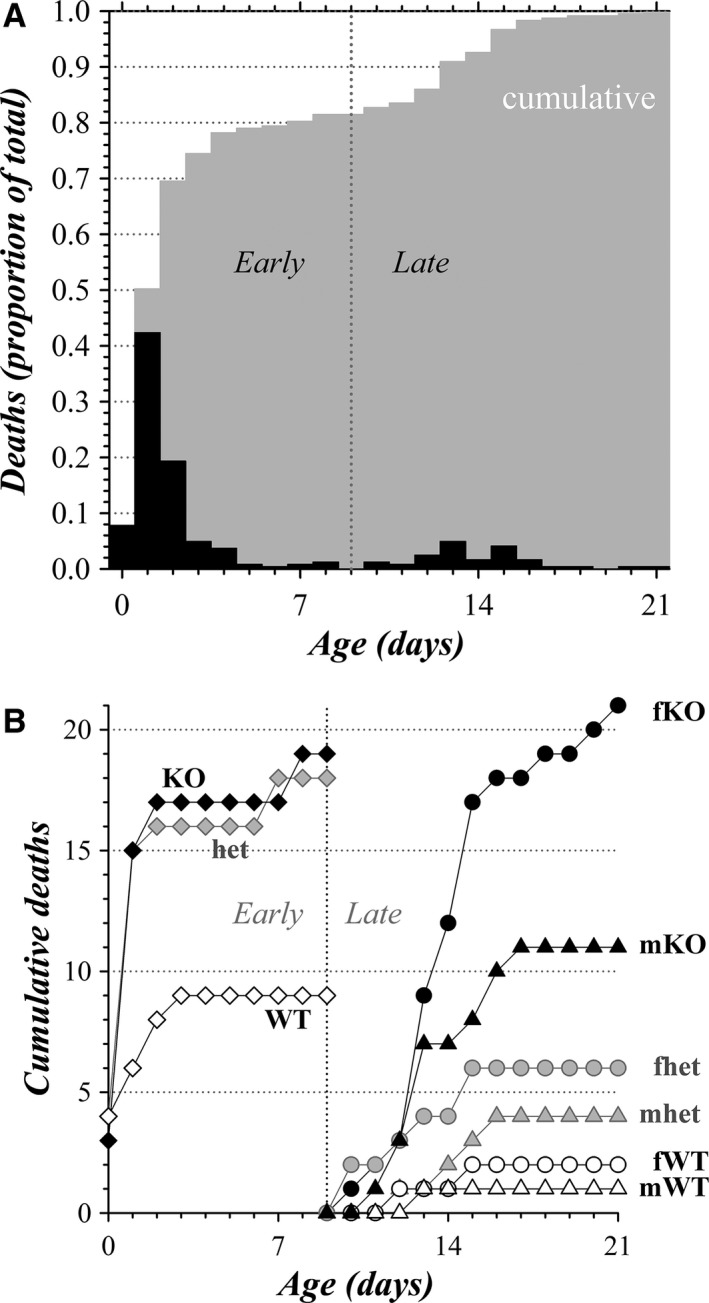
Pup mortality time course. (A) The frequency of pup death occurring at each postnatal age is plotted (black bars), together with the progression of total pup deaths at each day (*cumulative*, gray bars). A postnatal age of 9 days demarcates early and late periods of postnatal deaths (vertical dotted line). Of the 280 pup deaths, only those with a recorded day of death are shown (*n* = 243). Those undated deaths all occurred in the early period. (B) Cumulative pup deaths during early and late periods are plotted separately to aid comparisons among genotypes for the timing of death. Early period deaths (first 9 days, total genotyped sample, *n* = 46) are plotted at each postnatal age for genotyped pups (♢, WT; 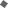, het; ♦, KO). Late period deaths (after 9 days, includes all late period deaths, *n* = 45) are shown at postnatal ages for each sex and genotype category (▵, mWT; ○, fWT; 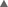, mhet; 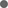, fhet; ▲, mKO; ●, fKO). See Table 2 for comparison with Mendelian expectation.

The age of pups found dead or noted as missing provided a time course of mortality during the postnatal nursing interval for all of the litters combined (Fig. 2A). These daily deaths accumulated during two separable phases (early and late), indicated by the intermediate plateau in cumulative deaths. Deaths most commonly occurred by 1 day of age (P1) with a smaller cluster occurring around the ages of P13 to P15. The onset of the late period deaths coincides with the developmental stage when pups acquire heightened sensory and motor capacities (Fox 1965; Smith et al. 2002; Branchi et al. 2003; Heyser 2004; Castelhano‐Carlos et al. 2010). Only a sample of the 235 pups dying before an age of 9 days were obtained for genotyping (19.6%), primarily dead pups recovered by LAR staff. KO pups contributed strongly to these deaths, supporting a non‐Mendelian distribution (Fig. 2B and Table 2). The full gender categorization for the late period deaths of nursing pups (Fig. 2B and Table 2) supported the likelihood that both mKO and fKO pups suffered a second episode of disadvantage before weaning. Although female pup deaths exceeded male deaths in each genotype category during the late period, this excess did not reach the statistical criterion of *P* ≤ 0.05 (29 females and 16 males, *χ* ^2^ = 3.76, *P* = 0.053), possibly due to the relatively small sample size.

**Table 2:** Pup attrition during maternal care

	First 9 days				
	WT	het	KO	$n_{p}^{d}$	*N*_L_
Observed	9	18	19	46	22
*Mendelian*	12	23	12		
Delta	−3	−5	+7		
*χ* ^2^	*0.54*	*1.09*	*4.89*	*6.52*,* P* = 0.038	

	After 9 days							
	mWT	fWT	mhet	fhet	mKO	fKO	$n_{p}^{d}$	*N*_L_
Observed	1	2	4	6	11	21	45	24
*Mendelian*	6	6	11	11	6	6		
Delta	−5	−4	−7	−5	+5	+15		
*χ* ^2^	*3.80*	*2.33*	*4.67*	*2.45*	*5.14*	*42.0*	*60.4*,* P* < 0.0001	

Genotype and sex of pups were determined for each litter (*N*_L_). Pups found dead prior to the standard genotyping period were collected for postmortem genotyping. The number of pups found dead (or missing after the date of genotyping) were compared with a Mendelian model based on the total number of dead pups ($n_{p}^{d}$). Individual categories deviating from this Mendelian model with a chi‐square larger than the value for *P* = 0.05 at one degree of freedom (3.84) are highlighted. The average number of pups born ($n_{p}^{b}$) for the group with early deaths was 9.2 ± 2.5 pups/litter and for late deaths was 9.9 ± 1.9 pups/litter (mean ± SD). Italics values indicate chi square values.

The clear loss of KO pups in excess of WT and het pups during nursing points to a weakness of the standard formulation for testing whether a genotype distribution conforms to Mendelian expectations (Table 1). By assuming that the total number of weaned pups corresponds to all of those in the sample, missing pups are ignored and the test produces a biased outcome. Because pups die during the nursing period (P0–P21), if the gender categories were known for all of these pups lost, then pup numbers could be added back to the distribution. However, a full categorization was lacking for early deaths, so that an alternative approach instead estimates the total pup number from a portion of the sample (see Methods). This approach supported the loss of mKO and fKO pups as a major source of departure from a Mendelian distribution along with fewer fhet pups (Table 1, *Mendelian2*), compared with the conventional approach that suggested an advantage for mWT pups.

**Figure 3:**
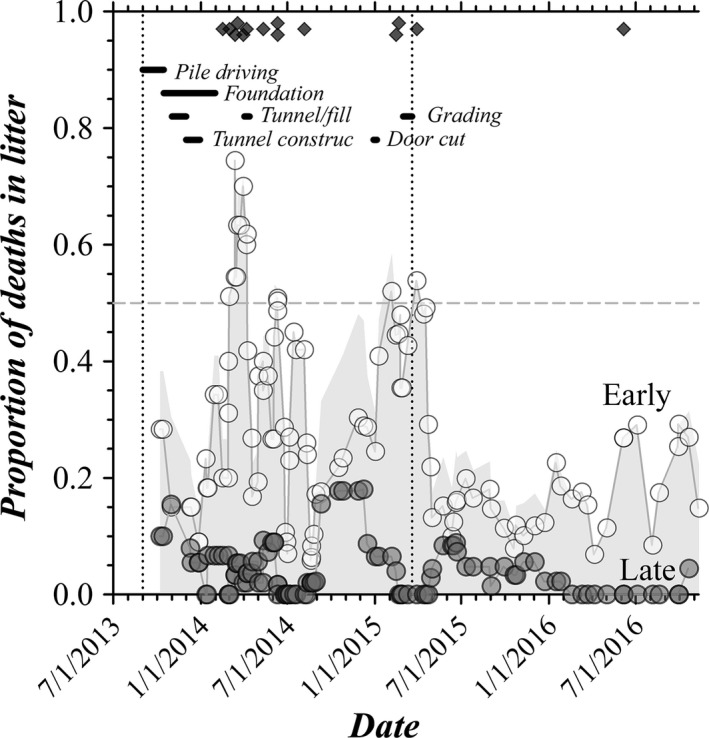
Chronology of pup survival. The proportion of deaths in WSU colony litters is shown as the running average (five litters) for deaths in the early postnatal period (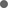), late postnatal period (○), and total (gray shaded area). The dates of total loss litters are indicated (♦, *n* = 14). The beginning and end of the construction period are indicated by vertical dotted lines with the timing of documented construction events indicated by black bars. Tunnel excavation was followed by tunnel construction and then by filling and compaction of the trench.

Pup survival improved dramatically coincident with the completion of construction for a building adjacent to the vivarium housing the BK colony, which defined two distinct periods of colony outcomes, construction and quiescent. Poor weaning success occurred episodically during the period of construction (Fig. 3) with failures of total litters generally following major construction events. The adjacent building is within 100 m of the animal housing site and the connector tunnel entrance is within 10 m of the room housing the BK colony. Direct assignment of construction as the cause of pup death was not apparent, as different litters gestating and nursing during the same construction events either suffered losses or were successful. The timing of litter loss with construction events did suggest a connection with multiple stressful episodes, such that greater losses may have occurred if the dam experienced stress as a fetus and then again during gestation of a first litter. For complete loss events, 10 (71%) of 14 were first litters for a dam.

The presumed stress associated with construction did not disturb all of the breeding outcomes. Although postnatal pup death decreased dramatically after construction ceased (Fig. 3), mean litter size was similar (quiescent, 8.6 ± 2.3 pups/litter, *N*_L_ = 37; construction, 8.0 ± 2.2 pups/litter, *N*_L_ = 73) as was the proportion of male pups (quiescent, 0.53 ± 0.04, *N*_L_ = 16; construction, 0.57 ± 0.04 pups/litter, *N*_L_ = 15; compare with Fig. 1B). Interestingly, the distribution of litter sizes was strongly bimodal in the quiescent period (modes of 7 and 9 pups), similar to the Donahue dataset for WT C57BL/6J mouse breeding (Fig. 1A). In comparison, the distribution of litter sizes during the construction period was unimodal (eight pups). The proportion of pup deaths during the late nursing period (P10–P21) also was similar in the quiescent period (0.041, 13 of 317 born) and during construction (0.055, 32 of 587 born) with a preponderance of KO deaths. The stretch of 16 consecutive litters without a late period pup loss during the quiescent period was similar to stretches of 15 and 13 consecutive litters without a late pup loss during construction, but also could presage an end to late period deaths in the absence of vibrational/noise stress.

**Figure 4:**
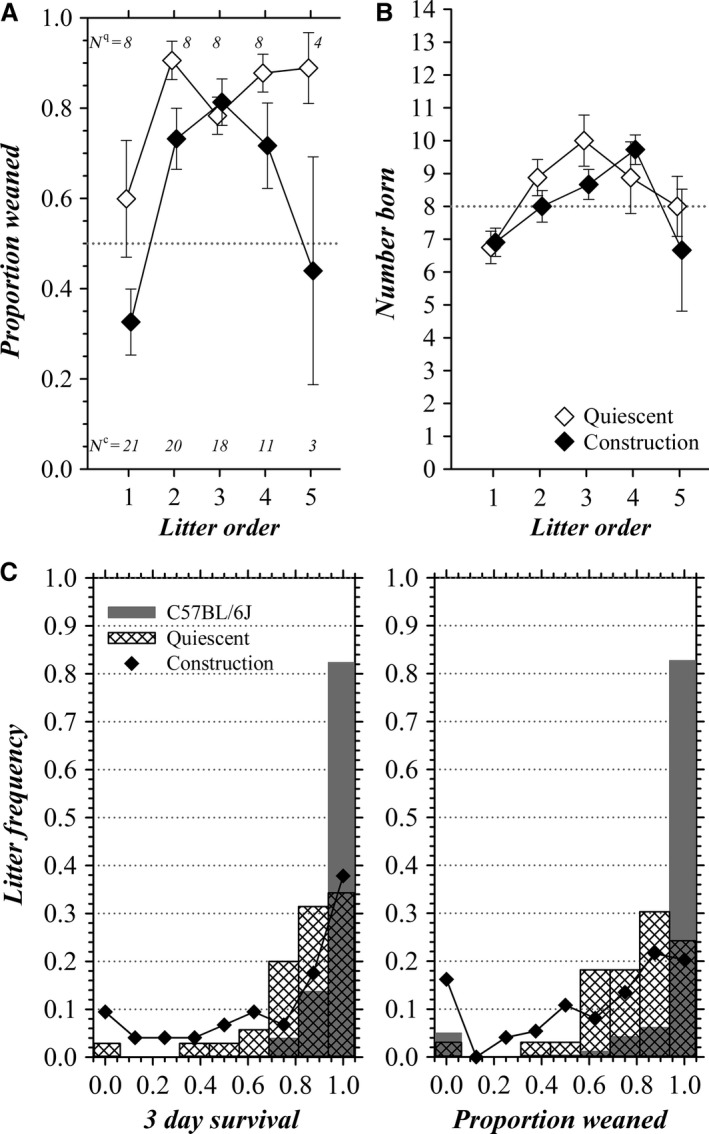
Litter success. The mean proportion of pups (all genotypes) weaned from litters are plotted (A) according to the litter order of dams, first through fifth (mean ± SEM); the mean number of pups (all genotypes) in a litter also are plotted (B). Litters born during the construction period (♦) are compared to litters born during the quiescent period (♢). The number of litters is shown, for quiescent litters (*N* ^q^) and construction litters (*N* ^c^). (C) The proportions of pups surviving in a litter are plotted as a frequency histogram for C57BL/6J breeding pairs (gray bars) overlain with quiescent period heterozygotic BK–null breeding pairs (transparent hatched bars), at 3 days of age in the left panel (*N*_L_ = 35, WSU colony; *N*_L_ = 51, Mouse Phenome Donahue19 dataset, reproduction‐fecundity, percent survived day 3, C57BL/6J; Donahue 2016) and at weaning in the right panel (*N*_L_ = 33, WSU colony; *N*_L_ = 260, Mouse Phenome Jax5 dataset, reproduction‐fecundity, weaned to born ratio, C57BL/6J; The Jackson Laboratory 2016). The heterozygotic BK–null breeding pair results during construction also are plotted (♦, *N*_L_ = 74).

A striking difference for the quiescent period compared with construction was the near absence of complete litter losses (Fig. 3). An association of complete litter losses with construction‐related stress supported earlier studies indicating that complete litter losses are rare and likely stemming from a husbandry issue separate from infanticide (Weber et al. 2013a, 2013b). Similarly, the proportion of early deaths (P0–P8) was lower for the quiescent period, 0.15 (47 of 317 born), compared with construction, 0.32 (188 of 587 born). First litters of dams exhibited lower pup survival (Fig. 4A); and, the number of pups in first litters also tended to be smaller than subsequent litters (Fig. 4B). Loss of entire litters by an age of 3 days during construction occurred at an incidence of ~9% and at an incidence ~16% by the time of weaning (Fig. 4C), greatly exceeding the results from documented C57BL/6J wild‐type breeding (Donahue 2016; The Jackson Laboratory 2016). The bias toward KO pup deaths during the early postnatal period was even more evident when considering only the quiescent period (4 WT, 14 het, 17 KO; Mendelian *χ* ^2^ = 11.06, *P* = 0.004), consistent with the depressed proportion of pups surviving within WSU colony litters compared with WT breeding (Fig. 4C). Presumably, the lower survival within litters resulted from deficiencies imposed by the absence of the BK channel.

Only two WSU colony mice died after weaning, both at an age of 16 weeks, a mWT mouse and a fKO mouse. The mWT mouse may have died from complications of a blocked urethra noted at necropsy. The fKO mouse was euthanized because of apparent hind limb paralysis, and at necropsy, one uterine horn was distended with fluid and the other contained blood. In addition, after euthanasia for tissue harvest, another fKO mouse (25 weeks of age) was noted to have both uterine horns distended by fluid. Thus, the total postweaning mortality was only 0.3% of weaned mice (2/624). In contrast to the BK^KO^ mice in this WSU‐C57BL/6J colony, BK^KO^ mice on an inbred FVB/NJ background exhibited 40% mortality occurring at 9.4 weeks of age (Meredith et al. 2004).

### Pup survival – prenatal

Death of pups before gender categorization limited the ability to determine whether WSU colony births followed Mendelian proportions (Table 3A). Because building construction also likely contributed stressful stimuli during gestation, breeding outcomes were examined in litters born during construction (from beginning of construction until 30 days after ending, *N* = 73) compared with litters born in the quiescent period (*N* = 37). The likely skew introduced into the list of genotyped mice due to pups that died prior to categorization was compensated in part by considering only litters with complete gender categorization. Limiting the comparison to these completely categorized litters reduced the sample size, but importantly also led to increased *χ* ^2^. Including only complete litters with ≥8 pups further reduced the sample size while minimizing the chance of errors due to pups missing from categorization. As considered for weaned mice (Table 1), using a Mendelian model based on the total number of mice born likely underestimated the cohort by ignoring mortality prior to birth. A second Mendelian model was calculated (Table 3B) assuming that the number of mWT and mhet pups represented a less biased estimate of the cohort for each litter (see Material and Methods). In the quiescent period, fKO pups apparently were disadvantaged prior to birth (largest contribution to the chi‐square), and unexpectedly fhet pups instead were disadvantaged during construction. The analysis of weaned pups (Table 1) indicated a deficit in fhet pups weaned, also supporting that this disadvantage occurred prior to weaning. These results together suggested that construction‐related stress altered the prenatal condition to disfavor fhet pups rather than fKO pups.

**Table 3:** (A) Pups genotyped and (B) pups genotyped – complete litters, ≥8 pups in litter

	mWT	fWT	mhet	fhet	mKO	fKO	$n_{p}^{g}$	*N*_L_
(A) Pups genotyped^a^								
Observed	107	93	182	164	80	90	716	98
*Mendelian*	90	90	179	179	90	90		
Delta	+17	+3	+3	−15	−10	0		
*χ* ^2^	*3.42*	*0.14*	*0.05*	*1.26*	*1.01*	*0.003*	*5.88*,* P *= 0.32	
				$n_{p}^{b}$ = 8.5 ± 2.0 pups born/litter				
(B) Pups genotyped – complete litters, ≥8 pups in litter^b^								
*Quiescent*								
Observed	23	18	44	39	19	13	156	16
*Mendelian*	20	20	39	39	20	20		
Delta	+3	−2	+5	0	−1	−7		
*χ* ^2^	*0.63*	*0.11*	*0.64*	*0.00*	*0.01*	*2.17*	*3.56*,* P* = 0.61	
*Mendelian2*	*22*	*22*	*45*	*45*	*22*	*22*	**179**	16
Delta	+1	−4	−1	−6	−3	−9		
*χ* ^2^	*0.02*	*0.85*	*0.01*	*0.74*	*0.51*	*3.93*	*6.06*	
				$n_{p}^{b}$ = 9.8 ± 1.3 pups born/litter				
*Construction*								
Observed	25	15	40	20	13	24	137	15
*Mendelian*	17	17	34	34	17	17		
Delta	+8	−2	+6	−14	−4	+7		
*χ* ^2^	*3.62*	*0.26*	*0.97*	*5.93*	*0.99*	*2.76*	*14.53*,* P* = 0.013	
*Mendelian2*	22	22	43	43	22	22	**174**	15
Delta	+3	−7	−3	−23	−9	+2		
*χ* ^2^	*0.49*	*2.09*	*0.28*	*12.69*	*3.52*	*0.23*	*19.31*	
				$n_{p}^{b}$ = 9.1 ± 1.2 pups born/litter				

a Genotype and sex of pups were determined for each litter (*N*_L_) and compared with a Mendelian model based on total pup number ($n_{p}^{g}$).

b Genotype and sex of pups were determined for each litter (*N*_L_). Complete litters were those lacking any observed attrition in pup number prior to genotyping. The number of genotyped pups were compared with a Mendelian model based on total pup number ($n_{p}^{g}$) or the number of mWT and mhet pups genotyped (bold, *Mendelian2*, see Methods). Individual categories deviating from these Mendelian models with a chi‐square larger than the value for *P* = 0.05 at one degree of freedom (3.84) are highlighted. Bold value indicates predicted total number based on the logic in methods. Italics values indicate chi square values.

**Figure 5:**
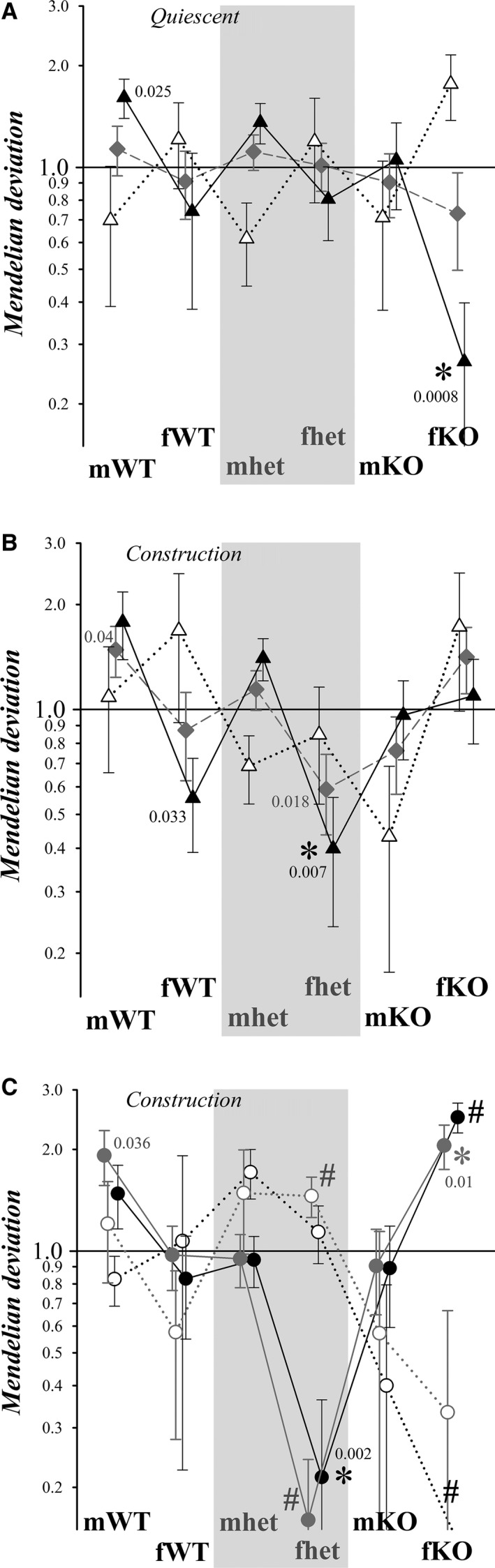
Deviations in genotype distribution from Mendelian proportions. The deviation from the expected Mendelian proportions for gender categories within litters was calculated as the ratio (observed/expected) such that a value of 1.0 indicated the presence of the expected Mendelian proportion (see Material and Methods). Values different from 1.0 (*P* ≤ 0.05) are indicated by a *P‐*value. (A) The Mendelian deviations (mean ± SEM) of complete litters (≥8 pups) born during the quiescent period (no construction) are plotted (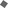, *N*_L_ = 16, mean ± SEM). Subgroups from these litters were defined based on the number of male pups: >4 male pups (▲, *N*_L_ = 8), <4 male pups (▵, *N*_L_ = 5). Bonferroni correction for multiple measures within each subgroup provided a statistical criterion of *P* ≤ 0.008, marked by asterisks. (B) The Mendelian deviations (mean ± SEM) of complete litters (≥8 pups) born during construction are plotted (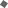, *N*_L_ = 15, mean ± SEM), as in panel A, along with subgroups defined by the number of male pups: >4 (▲, *N*_L_ = 8), <4 (▵, *N*_L_ = 4). (C) Subgroups were defined using selected female genotypes (marked by #) for litters born during construction: <2 fhet pups (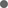, *N*_L_ = 8), >2 fhet pups (○, *N*_L_ = 3), >1 fKO pups (●, *N*_L_ = 7), <1 fKO pups (○, *N*_L_ = 4). Bonferroni correction for multiple measures within each subgroup provided a statistical criterion of *P* ≤ 0.01, marked by asterisks.

Assessment of deviations from a simple Mendelian model on a litter by litter basis provided a means to estimate variability for each gender category (see Material and Methods). In the quiescent period, gender categories of complete litters conformed to Mendelian expectations (Fig. 5A). Deviations from Mendelian expectations during construction (Fig. 5B) suggested depletion of fhet pups (0.59 ± 0.15). Previous studies established an intrauterine influence of male fetuses on adjacent female fetuses likely via testosterone (Ryan and Vandenbergh 2002). Because an ideally Mendelian litter of eight pups would have four males, these complete litters were sorted for those with greater than four males and for those with fewer than four males. The quiescent group of litters with >4 males exhibited a deficiency of fKO pups (0.27 ± 0.13) that reached a statistical criterion of *P* = 0.0008, while litters with <4 males otherwise resembled Mendelian expectations (Fig. 5A). Construction litters with >4 males instead indicated a lower than expected presence of fhet pups (0.40 ± 0.16, *P* = 0.007) and suggested a deficiency of fWT pups (Fig. 5B).

Possible prenatal interactions with fhet pups were examined further by sorting complete litters for <2 fhet pups and >2 fhet pups (2 fhet pups expected for a Mendelian litter of 8 pups). The group of construction litters (Fig. 5C) with <2 fhet pups exhibited a higher than expected contribution of fKO pups (2.05 ± 0.31, *P* = 0.01), whereas litters with >1 fKO pups exhibited fewer than expected fhet pups (0.21 ± 0.15, *P* = 0.002). In comparison, litters with >2 fhet pups or <1 fKO pup otherwise conformed to Mendelian expectations. A similar sorting was performed based on mhet and mKO pups as well as mWT and fWT pups. Only one of the deviations reached a statistical criterion of *P* ≤ 0.01, a deficit of fhet pups (0.29 ± 0.20, *P* = 0.009, *N*_L_ = 8) for litters with >2 mWT pups, consistent with an influence by male fetuses (Fig. 5B). For litters born during the quiescent period, this litter sorting generated one gender category exhibiting a deviation that reached the statistical criterion of *P* ≤ 0.01, a deficit of fKO pups (0.11 ± 0.11, *P* = 0.0002, *N*_L_ = 7) for litters with >2 mhet pups, again consistent with an influence by male fetuses (Fig. 5A). Thus, prior to birth, the absence of the BK channel appeared disadvantageous primarily to fKO pups with presumed adjacent male fetuses. Together, these results suggest that construction‐related stress shifted intrauterine interactions such that the presence of fKO pups increased fhet pup mortality.

### Mouse growth

**Figure 6:**
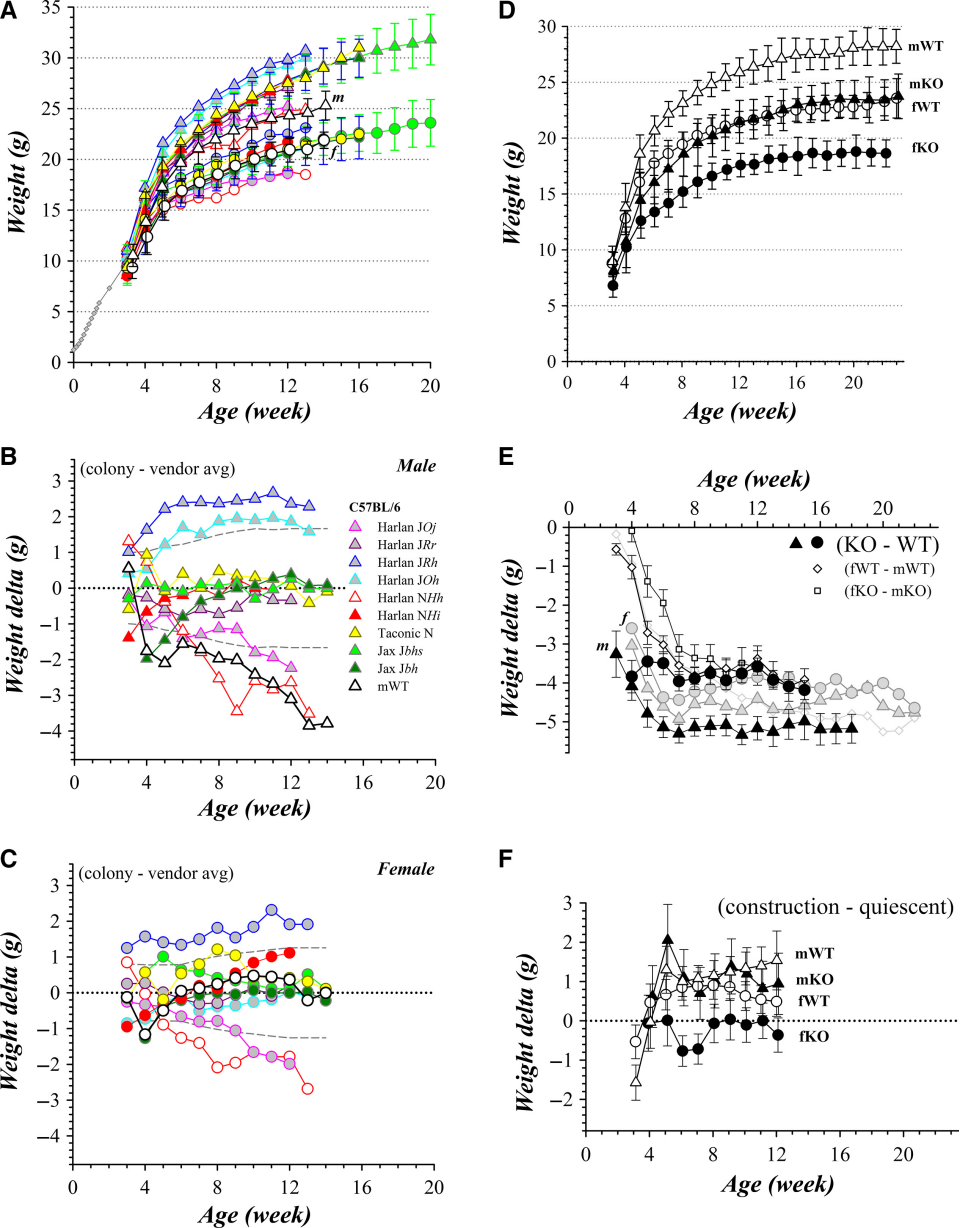
Growth of colony mice: weight gain after weaning. (A) Weekly weights of quiescent period WT mice are plotted (▵, male; ○, female; mean ± SD). The census varied from week to week with the number of mice generally declining over the period from 28 to 10 for males and from 20 to 15 for females. Weights of nursing pups from previous studies are plotted (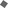; C57BL/6N; Ucar et al. 2010; C57BL/6; Palmer et al. 2006). Growth curves for mice (triangles, male; circles, female; mean ± SD) from nine commercial vendor sources also are plotted: The Jackson Laboratory C57BL/6J, Stock Number 000664, LabDiet 5K52/5K67 (filled light green, *N* = 450, Production Facilities, Bar Harbor, ME and Sacramento, CA, USA, https://www.jax.org/jax-mice-and-services/strain-data-sheet-pages/body-weight-chart-000664; filled dark green, *N* = 100, Production Facilities Bar Harbor, ME, USA, December 2007, http://jackson.jax.org/rs/444-BUH-304/images/physiological_data_000664.pdf); Envigo Biosciences/[Harlan], Teklad Global Rodent Diet 2018S (filled red, C57BL/6N*Hsd*,* N* = 100, Production Facility 217, Indianapolis, IN, USA, 2011; open red, C57BL/6N*Hsd*, Production Facility 505‐B2, Horst, the Netherlands, 2015; cyan/gray, C57BL/6J*OlaHsd*, Production Facility 505‐B2, Horst, the Netherlands, 2013; pink/gray, C57BL/6J*OlaHsd*, Production Facility 610, Jerusalem, Israel, 2015; blue/gray, C57BL/6J*RccHsd*, Production Facility 505‐B2, Horst, the Netherlands, 2014; purple/gray, C57BL/6J*RccHsd*, Production Facility 640, Rehovot, Israel, 2015; http://www.envigo.com/products-services/research-models-services/models/research-models/mice/inbred/c57bl-6-inbred-mice/); Taconic Biosciences C57BL/6N*Tac*, NIH 31‐M Rodent Diet (filled yellow, *N* = 500, 2013, http://www.taconic.com/mouse-model/black-6-b6ntac). (B, C) The age‐related weight differences for each colony compared with the mean of commercial vendor sourced mice (delta = colony – vendor avg) are plotted for male and female WT mice (symbols the same as in panel A); together with the standard deviation of the vendor mean (gray dashed lines). (D) The weekly weights of construction period WT and BK^KO^ mice are plotted (▵, male wild type, mWT; ○, female wild type, fWT, ▲, male knockout, mKO; ●, female knockout, fKO; mean ± SD). The census varied from week to week with the number of mice ranging from 53 to 14 for mWT and fWT and from 36 to 15 for mKO and fKO. The weights of mhet and fhet mice were indistinguishable from the respective WT mice (data not shown). (E) The age‐related weight differences between KO and WT mice are plotted (KO‐WT), both as the difference between group means of construction period mice (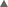, male; 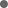, female) and means of all littermate differences (▲, male, *n* = 37; ●, female, *n* = 31; mean ± SEM). The group mean difference between fWT and mWT (♢) also are plotted along with the means of littermate differences for fWT‐mWT (♢, *n* = 41, mean ± SEM) and fKO‐mKO (□, *n* = 32, mean ± SEM). (F) The age‐related weight differences between mice from the construction and quiescent periods (construction − quiescent) are plotted (▵, mWT; ○, fWT, ▲, mKO; ●, fKO; mean ± SEM). The SEM for these differences was calculated using the SEM values of the construction and quiescent groups (√(SEM_C_^2^ + SEM_Q_^2^); Bevington and Robinson 2003). The mean of delta weights for week 6 through week 10 were calculated and compared with an expectation of not different from zero (mWT, +1.09 ± 0.06 g, *P* = 0.00006; mKO, +1.08 ± 0.11 g, *P* = 0.0007; fWT, +0.83 ± 0.05 g, *P* = 0.00007; fKO, −0.32 ± 0.17 g, *P* = 0.13; *n* = 5, mean ± SEM).

WSU colony WT mice grew rapidly after weaning with a relative plateau at ~15 weeks; those from the quiescent period are shown in Figure 6A. Similar C57BL/6 mice available from commercial suppliers attain different weights after this initial postweaning period. These differences are not easily attributable to substrain, diet, or facility location. Interestingly, the largest WT C57BL/6J male mice are raised at a facility in Horst, the Netherlands at which WT C57BL/6N male mice have the smallest weights of the comparison group; this growth pattern is followed only partially by the female mice at this facility. A clearer focus on the differences among the colonies is accomplished by subtracting the mean for commercial mice (Fig. 6B and C). The initial growth spurts after weaning (week 4) were smaller for both the WSU colony mWT (Fig. 6B) and fWT (Fig. 6C) mice than for the mean of commercially raised mice. The mWT mice maintained this deficit in weight during subsequent weeks (>1 SD below the commercial mean), attaining ~4 g less weight at 15 weeks compared with the most similar C57BL/6J male mice (Jax, Fig. 6B). The fWT mice grew at rates nearly identical to commercially available C57BL/6 female mice at times longer than 6 weeks with a tendency toward slightly higher weight at ages between 8 and 12 weeks (Fig. 6C).

WSU colony mKO and fKO mice attained lower weights than respective WT mice throughout the postweaning period; those from the construction period are shown in Figure 6D. Group means indicated an ~4 g lower weight for KO mice than for WT mice whether from the quiescent or construction period (Fig. 6E). Littermate comparisons further supported this growth deficit with fKO maintaining a 4‐g deficit from 7 to 15 weeks and mKO a 5‐g deficit from 7 to 18 weeks. The WT mice also exhibited a weight sexual dimorphism, with the fWT ~1 g lighter than mWT at 4 weeks that further separated to an ~3.5 g deficit by 7 weeks and continuing through 16 weeks of age (Fig. 6E). Strikingly, the sexual dimorphism for WSU colony WT mice was smaller than any commercial colony, and roughly half the mean dimorphism of –6.8 ± 0.8 g (fWT − mWT; mean ± SD) exhibited by commercial mice at 12 weeks of age.

**Figure 7:**
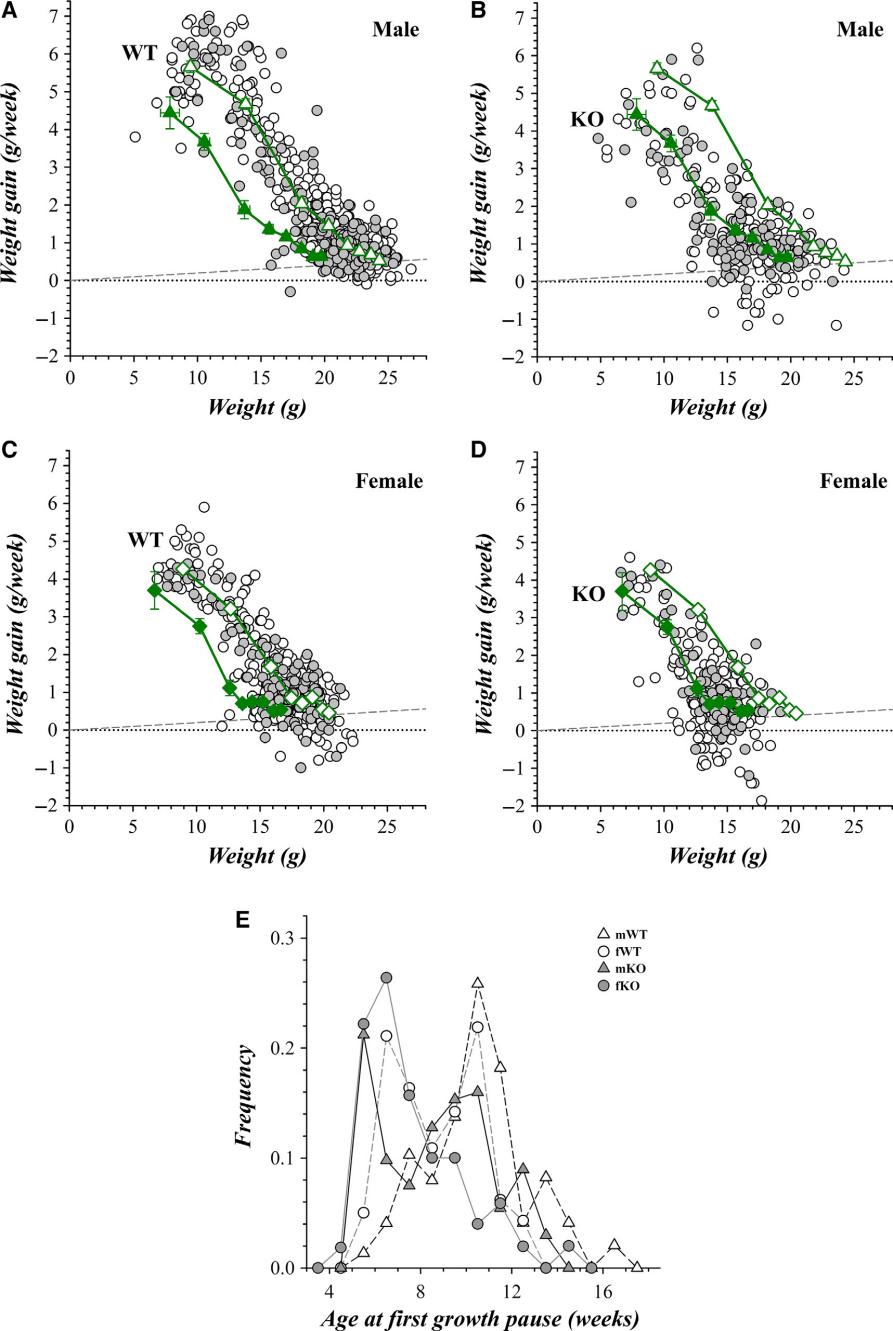
Weekly weight gain of colony mice. The weekly weight gain with respect to the initial weekly weight is plotted (○, construction period; 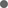, quiescent period) from week 3 to week 9 for each mouse. (A) mWT,* n* = 58 and 28; (B) mKO,* n* = 34 and 18; (C) fWT,* n* = 40 and 20; (D) fKO,* n* = 39 and 14. Lines connecting the values for individual mice were omitted for clarity. The weekly means of these weight gains also are plotted (mean ± SEM) beginning at week 3: mWT (A and B, green ▵, *n* = 70), mKO (B and A, green ▲, *n* = 43), fWT (C and D, green ♢, *n* = 46), fKO (D and C, green ♦, *n* = 42). The dashed lines represent a weekly weight gain of 2%. (E) The frequency of mice experiencing a first growth pause (weekly weight gain <2% of beginning weight) are plotted at the week of first pause occurrence (mWT, ▵, *n* = 55; fWT, ○, *n* = 53; mKO, 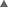, *n* = 39; fKO, 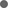, *n* = 52). Both quiescent and construction period pauses were combined because the distributions were similar. The proportion of mice experiencing a first growth pause by 9 weeks of age was 0.30 for mWT, 0.61 for mKO, 0.55 for fWT, and 0.88 for fKO.

Individual mice generally gained weight each week. Weekly weight gains not only progressively declined as the mouse reached adult age (Fig. 7), but also exhibited weeks with low weight gain or losses followed by resumed growth. Onset of these pauses in growth (<2% gain) occurred at ~20 g for mWT and ~15 g for fWT mice, corresponding to ages of ~6 weeks for males and ~5 weeks for females. The distributions of age at first growth pause were bimodal (6–7 and 10–11 weeks) with the dominant mode for mWT at 10–11 weeks (Fig. 7E). These earlier groups of pauses correspond with the weeks when the largest increase in weight separation occurred between the sexes (Fig. 6E). Both mKO and fKO mice exhibited earlier growth pauses with greater declines, but generally resumed weight gains the following week. These growth pauses did not appear to be related to general stresses within the vivarium, as pauses of cage mates and litter mates were not synchronous. Both the weight attained as well as the growth rate and the timing of pauses was similar for mice during and after construction.

**Figure 8:**
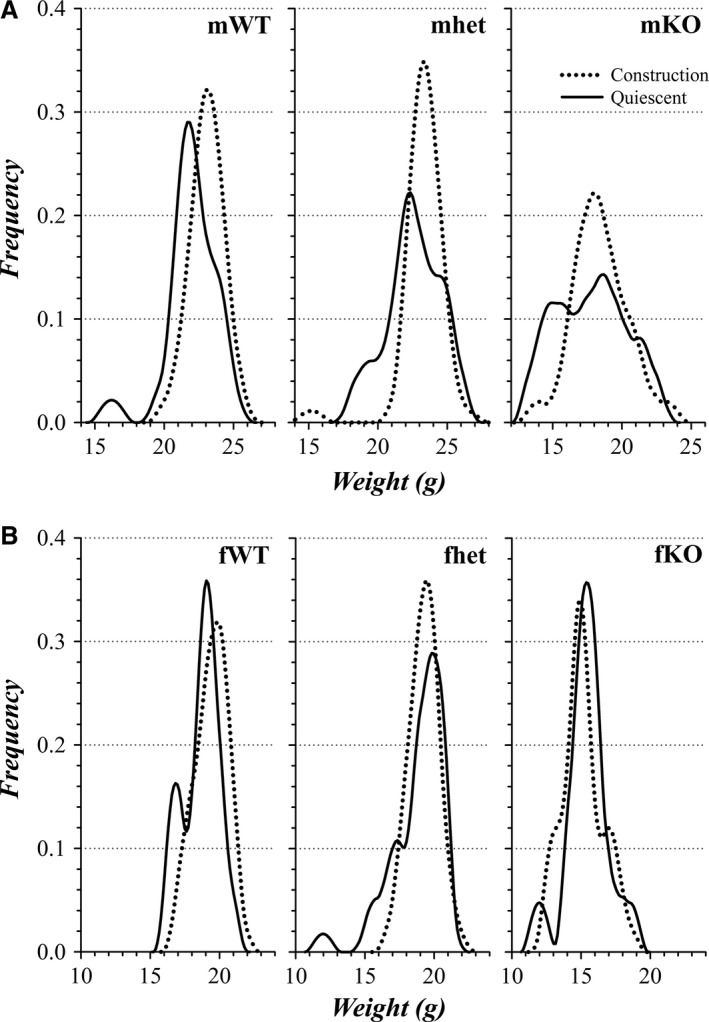
Weight distributions of colony mice: influence of construction. Weight distributions were generated by an average shifted histogram procedure followed by smoothing (see Material and Methods). (A) The distribution of weights for male mice at an age of 8 weeks during the quiescent period (solid line) and during the construction period (dotted line) are plotted (quiescent: *n* ^mWT^ = 26, *n* ^mhet^ = 34, *n* ^mKO^ = 19; construction: *n* ^mWT^ = 51, *n* ^mhet^ = 50, *n* ^mKO^ = 28). (B) The distribution of weights for female mice at an age of 8 weeks during the quiescent period (solid line) and during the construction period (dotted line) are plotted (quiescent: *n* ^fWT^ = 21, *n* ^fhet^ = 41, *n* ^fKO^ = 16; construction: *n* ^fWT^ = 31, *n* ^fhet^ = 46, *n* ^fKO^ = 31).

Although the weight difference between KO and WT was similar for mice from the quiescent and construction periods, mWT, mKO, and fWT mice appeared persistently ~1 g heavier when raised during the construction period compared with those same categories raised in the quiescent period (Fig. 6F). The distributions of weight at 8 weeks of age suggested the presence of subgroups within each category (Fig. 8). Rather than a simple shift of a unimodal distribution, the slightly higher weight of mice during the construction period appeared to result from a higher proportion of mice occurring in the larger subgroup.

**Figure 9:**
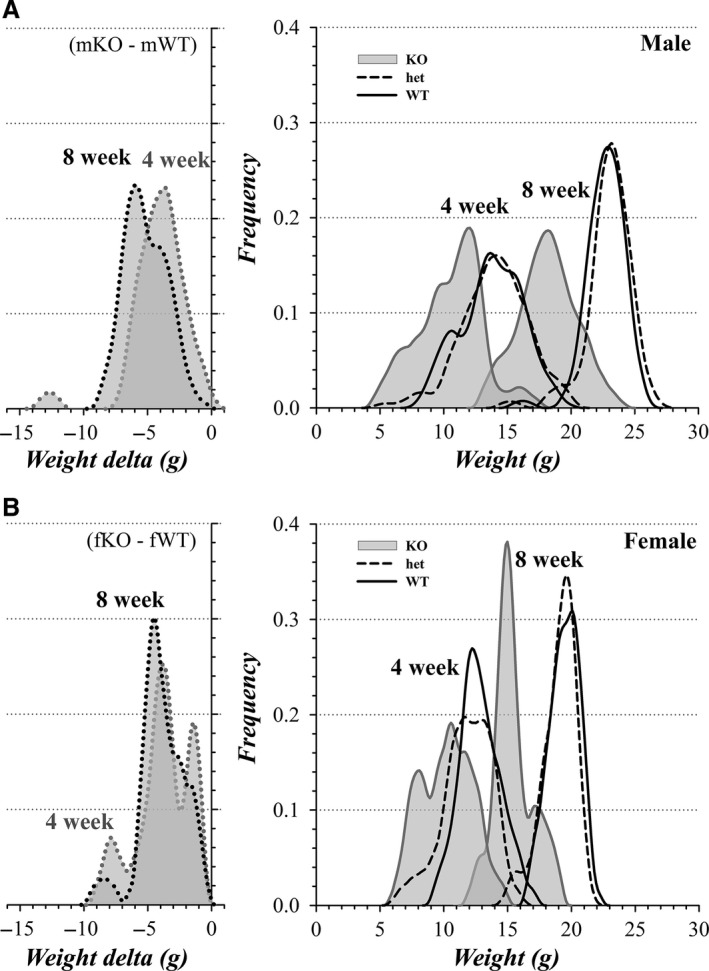
Weight distributions of colony mice: littermate comparison. Weight distributions were generated by an average shifted histogram procedure followed by smoothing (see Material and Methods). (A) The distribution of weight differences (delta) between littermate mKO and mWT are plotted at mouse ages of 4 weeks (gray dotted line, *n*_pair_ = 28) and 8 weeks (black dotted line, *n*_pair_ = 37). The distribution of weights for mWT (black line), mhet (black dashed line), and mKO (gray line) are plotted at mouse ages of 4 weeks (*n* ^WT^ = 59, *n* ^het^ = 73, *n* ^KO^ = 27) and 8 weeks (*n* ^WT^ = 53, *n* ^het^ = 56, *n* ^KO^ = 33). (B) The distribution of weight differences (delta) between littermate fKO and fWT are plotted at mouse ages of 4 weeks (gray dotted line, *n*_pair_ = 18) and 8 weeks (black dotted line, *n*_pair_ = 29). The distribution of weights for fWT (black line), fhet (black dashed line), and fKO (gray line) are plotted at mouse ages of 4 weeks (*n* ^WT^ = 34, *n* ^het^ = 51, *n* ^KO^ = 22) and 8 weeks (*n* ^WT^ = 33, *n* ^het^ = 46, *n* ^KO^ = 35).

The distribution of weights further reinforced the indication that KO mice attain lower weights than WT mice (Fig. 9). Focusing on littermate comparisons, every mKO mouse lagged behind their mWT littermates (Fig. 9A, every littermate paired delta was negative, mKO − mWT). By an age of 8 weeks, a subgroup of mKO mice lagged even further behind their littermates. At 4 weeks, the weight distributions appeared to contain several subgroups, but for mWT and mhet mice these differences became less noticeable by 8 weeks as indicated by the narrower distributions. The wide weight distribution during rapid growth occurring at 4 weeks may result in part from including mice born on different days of the week into a single midweek weighing. This age‐related variability appeared minimal at 4 weeks, because the standard deviation of the full mWT group was 2.31 g (*N* = 59) and that for littermate comparisons within the mWT group was 2.38 g (*N*_pair_ = 32). For mKO mice, the wide weight distribution persisted at 8 weeks suggesting that some mKO mice experience a stronger growth suppression that continued to restrain weight gain, unlike the tightening weight uniformity for mWT and mhet mice. Similarly, fKO mice lagged behind their littermate fWT mice, with wider distributions at 4 weeks than at 8 weeks (Fig. 9B).

**Figure 10:**
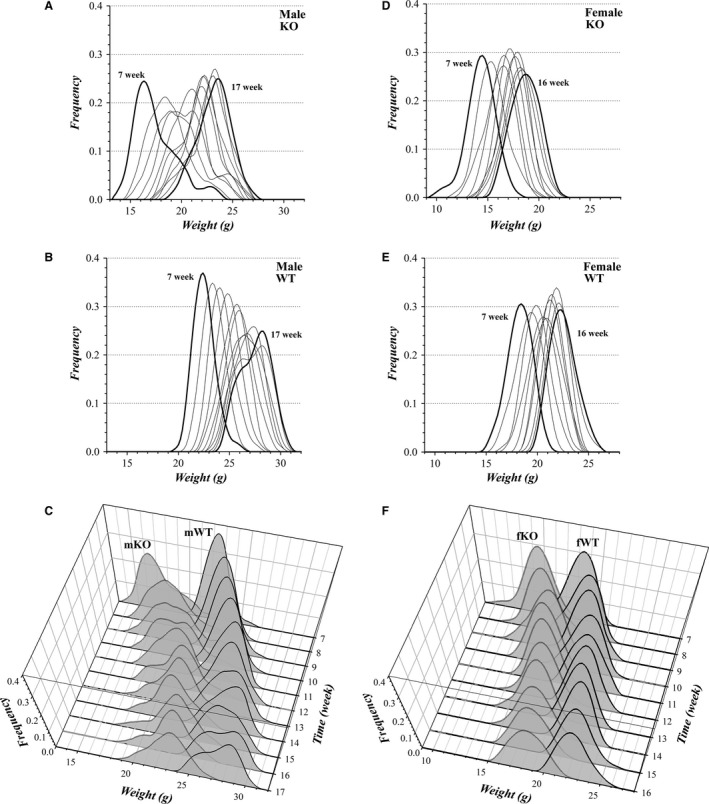
Weight distribution time course for colony mice. The same cohorts of male mice, mKO (A and C, *n*_m_^KO^ = 19), mWT (B and C, *n*_m_^WT^ = 23) were followed from week 7 to week 17, and for the cohorts of female mice, fKO (D and F, *n*_f_^KO^ = 17), fWT (E and F, *n*_f_^WT^ = 15) from week 7 to week 16. The sequential weekly weight distributions (average shifted histograms) are overlain (A, B, D, and E) with the first and last week in bold. The time course of these weight distributions is shown to illustrate the temporal variations in weight gain (C and F). Panels A and B are the *x*–*z* projections of panel C; and panels D and E are the *x*–*z* projections of panel F.

Tracking a cohort of mice showed that these weight distributions likely contain distinct subgroups that diverge and reconverge in weight over time (Fig. 10). The mKO category appeared to include a small group of individuals that attained greater weight earlier than the majority, and over 10 weeks the smaller mKO mice approached the size of this outlier mKO group (Fig. 10A, C). Interestingly, mWT mice diverged over the same period to exhibit two apparent weight subgroups (Fig. 10B, C). Littermate mWT mice generally tracked toward the same weight in the distribution, but littermate mKO mice occasionally were spread into distinct portions of the distribution. The fKO mice (Fig. 10D, F) and fWT mice (Fig. 10E, F) maintained narrower distributions than mKO and mWT mice, but the tails of the distributions and the variation in peak values indicated a similar converging and diverging of subgroups within these fWT and fKO populations.

**Figure 11:**
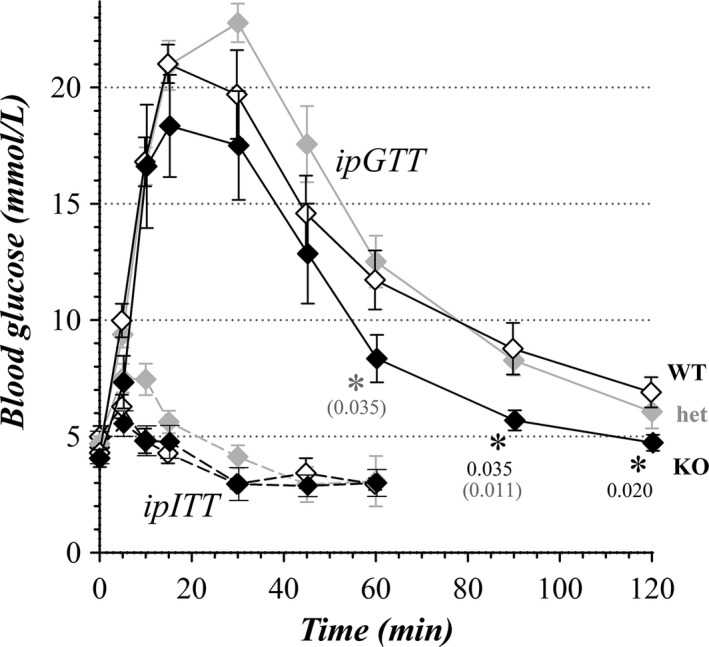
General glucose metabolism of colony mice. Time courses of blood glucose after intraperitoneal administration of glucose (solid lines, ipGTT) or insulin (dashed lines, ipITT) are plotted for WT (♢, *n* = 8), het (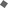, *n* = 7), and KO (♦, *n* = 6) mice. Results were from mice 6 to 7 weeks of age with male and female mice combined, because the results for sexes in each genotype were indistinguishable (*P* = 0.29). These glucose measurements were obtained during the hiatus in the middle of the construction period (Fig. 3). Glucose concentrations for KO mice significantly different from WT mice are indicated with an asterisk and *P* value, differences in KO from het indicated with *P* value in parentheses. The initial fasted blood glucose concentrations for KO and het mice were not significantly different than for WT mice (WT, 4.64 ± 0.36 mmol/L; het, 4.83 ± 0.39 mmol/L, *P* = 0.73; KO, 4.29 ± 0.56 mmol/L, *P* = 0.61; mean ± SEM). Areas under the curve of ipGTT results (calculated by trapezoid method without baseline subtraction; Andrikopoulos et al. 2008; McGuinness et al. 2009) for het and KO mice were not significantly different from those for WT mice (AUC^WT^ = 1.481 ± 0.127 mole·min/L; AUC^het^ = 1.570 ± 0.077 mole·min/L, *P* = 0.67 vs. WT; AUC^KO^ = 1.180 ± 0.123 mole·min/L, *P* = 0.17 vs. WT,* P* = 0.013 vs. het).

Maintenance of body weight involves influences from glucose metabolism. Responses of WSU colony mice to glucose and insulin tolerance tests (ipGTT and ipITT) indicated a largely similar regulation of blood glucose concentration among categories (Fig. 11). The lower blood glucose levels at 90–120 min for KO mice compared to WT mice suggested a more robust uptake of glucose possibly due to higher release of insulin. The early transient increase of blood glucose during ipITT likely resulted from handling induced stress; but, the stable suppression of blood glucose between 45 and 60 min during ipITT indicated similar overall tissue sensitivity to insulin for all categories of mice.

### Body composition

Determination of body composition into lean, fat, total water, and free water components by quantitative magnetic resonance provided a means to distinguish possible global metabolic distinctions among the gender categories. Normalizing these weight components to the total body mass allowed a direct comparison between WT mice and the smaller KO mice. The mass proportion for each component was stable over consecutive weekly measurements for ages between 6 and 10 weeks (data not shown). Derived comparisons of these components provided further indicators of body composition, specifically total proportion and hydration ratio (Table 4). Previous MRI determinations of fat proportion for male C57BL/6J mice, 0.074 ± 0.023 (Mathes et al. 2011; Mathes 2016) and 0.106 ± 0.049 (Bennett et al. 2016) (mean ± SD), were similar to the mWT mice in this study.

**Table 4:** Body composition of colony mice

	mWT	mhet	mKO	fWT	fhet	fKO
Weight	22.0 ± 1.0	22.9 ± 2.0	17.5 ± 2.4	18.4 ± 1.7	18.5 ± 1.7	15.5 ± 1.3
Lean proportion	0.859 ± 0.011	0.857 ± 0.013 (0.99)	0.838 ± 0.012 (0.0002)^a^	0.821 ± 0.009 (<0.0001)^a^	0.825 ± 0.011 (0.99)	0.808 ± 0.023 (0.11)
Fat proportion	0.074 ± 0.010	0.076 ± 0.010 (0.67)	0.095 ± 0.010 (<0.0001)^a^	0.097 ± 0.008 (<0.0001)^a^	0.092 ± 0.010 (0.67)	0.121 ± 0.017 (0.0002)^b^
Total H_2_O proportion	0.739 ± 0.012	0.737 ± 0.012 (0.73)	0.727 ± 0.014 (0.12)	0.722 ± 0.010 (0.006)a	0.732 ± 0.012 (0.17)	0.709 ± 0.024 (0.22)
Free H_2_O proportion	0.007 ± 0.003	0.005 ± 0.003 (0.51)	0.005 ± 0.004 (0.28)	0.006 ± 0.002 (0.99)	0.006 ± 0.004 (0.99)	0.006 ± 0.004 (0.99)
Total	0.941 ± 0.009	0.939 ± 0.011 (0.99)	0.938 ± 0.011 (0.99)	0.924 ± 0.008 (0.002)^a^	0.924 ± 0.016 (0.99)	0.935 ± 0.007 (0.49)
Hydration Ratio	0.852 ± 0.005	0.853 ± 0.007 (0.99)	0.862 ± 0.010 (0.003)^a^	0.872 ± 0.005 (<0.0001)^a^	0.880 ± 0.009 (0.03)	0.870 ± 0.009 (0.99)
*n*	15	20 [19]	15 [18]	9 [7]	15 [9]	9 [5]

The mean ± SD of body composition components are tabulated for the six gender categories. The “Total” value is the sum of lean, fat, and free water proportions. The “Hydration ratio” is the difference between total water and free water divided by lean ([tH_2_O − fH_2_O]/Lean). A mixed ANOVA model that included litter effects together with a stepdown Bonferroni multiple comparison procedure provided a comparison at a statistical criterion of *P* ≤ 0.008 (*P* values in parentheses: a, compared with mWT, dark gray highlight; b, compared with fWT, light gray highlight). The number of mice is indicated (*n*) with the number of litter mate pairs with WT in brackets.

**Figure 12:**
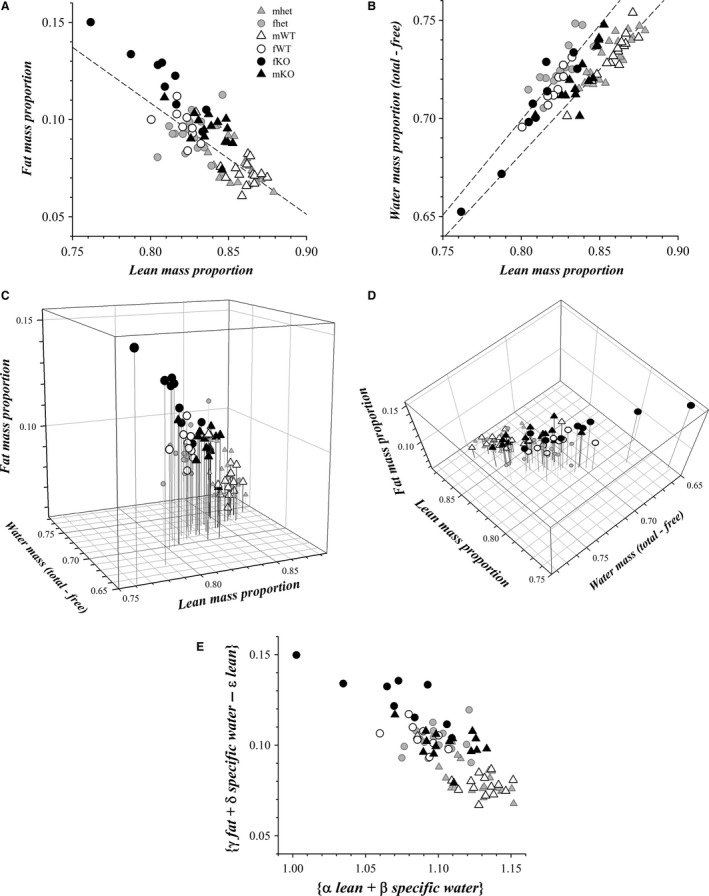
Body composition of colony mice. The components of body composition determined by EchoMRI Analyzer are plotted for mWT (▵, *n* = 15), mhet (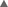, *n* = 20), mKO (▲, *n* = 15), fWT (○, *n* = 9), fhet (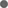, *n* = 15), fKO (●, *n* = 9) mice. These body composition measurements were obtained primarily after cessation of construction (mWT, 12 of 15; mhet, 16 of 20; mKO, 12 of 15; fWT, 6 of 9; fhet, 12 of 15; fKO, 7 of 9). Differences between these time period groups were not apparent. (A) The fat mass proportion is plotted in relation to the lean mass proportion along with a linear fit to the results from mWT and fWT mice (slope = −0.573, *x*‐intercept = +0.989, *y*‐intercept = +0.567, *r* ^2^ = 0.737). (B) The specific water mass proportion (Total − Free) is plotted in relation to the lean mass proportion along with a linear fit to the results from mWT and mhet mice (slope = +0.861, *x*‐intercept = +0.008, *y*‐intercept = −0.007, *y*‐[*x*→1.0] = +0.854, *r* ^2^ = 0.799) as well as for fWT and fhet mice (slope = +0.969, *x*‐intercept = +0.078, *y*‐intercept = −0.076, *y*‐[*x*→1.0] = +0.893, *r* ^2^ = 0.684). (C) The lean mass proportion {*x*}, fat mass proportion {*z*}, and specific water mass proportion (Total − Free) {*y*} are rendered as a 3D plot (symbols as in panels A and B). Panel A is the *x*–*z* projection, and panel B is the *x*–*y* projection of panel C. (D) Rotation of the 3D plot to the perspective from above and slightly behind brought the mWT and fWT points into alignment, supporting the existence of a surface within lean–fat–water space describing body composition for C57BL/6J mice. This rotation was optimized by least squares for the values from mWT, fWT, mhet, and fhet mice (rotation around the *z*‐axis by 39° [*φ*] and around the resulting *x*'‐axis by 21.5° [*ψ*]). (E) The view of this surface (seen edge on in panel D) was defined by the set of rotated axes for this plane of projection: *x*'‐axis = α lean + β specific water, α = cos *φ* ≅ 0.777, β = sin *φ* ≅ 0.629; *z*'‐axis = γ fat + δ specific water − ε lean, γ = cos *ψ* ≅ 0.930, δ = αθ ≅ 0.285, ε = βθ ≅ 0.231; and the orthogonal *y*’‐axis = ζ specific water − η lean − θ fat, ζ = αγ ≅ 0.723, η = βγ ≅ 0.586, θ = sin *ψ* ≅ 0.366. Any point in this plane (*y*’ = 0) can be transformed to the measurable values: lean = *x* = α *x*’ − ε *z*’; fat = *z *= γ *z*’; specific water = *y *= β *x*’ + δ *z*’.

Comparison of the fat mass proportion and lean mass proportion exhibited the expected inverse relationship (Fig. 12A). Clustering of the gender categories occurred, illustrating the statistical distinctions obtained from littermate comparisons (Table 4). The linear fit for mWT and fWT mice also provided an indication of internal consistency with an *x*‐intercept of 0.99, the lean proportion at zero fat. Additional clustering of gender categories occurred for comparison of specific water mass (Total − Free) proportion with lean mass proportion (Fig. 12B). Linear fit to the mWT and mhet values was distinct from the fit to fWT and fhet, supporting an apparent difference between male and female mice with regard to water handling, which concurred with the higher hydration ratio for fWT mice (Table 4). In addition, the fits supported internal consistency because the *x*‐intercepts of 0.01 and 0.08, respectively, conform to expectations that specific water mass occurred as a constituent of lean mass. The shift to fat over lean for KO mice compared with WT mice (Fig. 12A) seemed to contradict naïve expectations arising from the lower adult weights of KO mice (Figs. 6 and 9).

Rotation of the 3D plot for these body composition components (Fig. 12C and D) extended the demonstration of clustering to suggest the presence of an oblique surface within lean–fat–water space that defines distinct composition types. A plane approximating this surface provided axes that interconnect the lean, fat, and water measures of body composition (Fig. 12E). These new *x*’ and *z*’ axes define a plane containing the body composition types for both male and female mice. This roughly planar distribution implies that deviations along the *y*’ coordinate were physiologically minimized (ζ specific water − η lean − θ fat ≈ 0, Fig. 12E). A fatter and wetter composition type distinguished fWT from mWT mice, and mKO adopted a type more comparable with fWT mice. The most extreme departure occurred for fKO mice with an even fatter composition type. This 3D fit of the data is not unique, but does obey the constraint that these two rotations of the axes preserve the origin.

## Discussion

Proteins generated as gene products within cells contribute to physiologic activities maintaining cell vitality that influence associated tissues, organs, and ultimately the whole organism. Some proteins may be indispensable, leading to lethality when the protein is absent, with other proteins contributing to adaptations helpful during stress, but otherwise providing functions replaceable by other proteins (Turgeon and Meloche 2009; Bolon 2015). During development, these influences also likely wax and wane leading to critical times for that protein to function (Papaioannou and Behringer 2012; Bolon and Carreira 2015; Bolon and Ward 2015). The presence of the BK channel protein in many cells types (Contreras et al. 2013) creates the possibility for numerous points of altered function when this protein is absent, as illustrated both overtly and covertly in BK^KO^ mice. The overall viability of BK^KO^ mice (Meredith et al. 2004; Sausbier et al. 2004) indicates that opening this channel must only serve to adapt cellular responses for optimal performance, such that many of the deficits have a subtle presentation. For vivarium‐housed mice that do not encounter a full set of environmental stresses, the consequences of lacking BK channels in some cell types likely go unnoticed in BK^KO^ mice.

### Influences of construction vibration/noise

Building construction near animal holding rooms creates noises and vibrations in excess of those typically associated with vivarium maintenance (Castelhano‐Carlos and Baumans 2009; Rasmussen et al. 2009), which may lead to deleterious outcomes for the resident animals (Dallman et al. 1999). A daily 6‐min bout of recorded jackhammer noise is sufficient to reduce mouse litter size by 43% when it occurs during the period of blastocyst implantation (Rasmussen et al. 2009). The incidence of stillborn pups increases from a control value of 0.4 per 100 pups born to 3–4 per 100 pups born when this noise occurs during the first through third week of gestation. In comparison, this noise stress fails to alter postnatal weight gain from P0 through P7. Vibrations transmitted to animals will deliver maximal force where the motion is unattenuated by an object, which occurs at the resonance frequency range (RFR) specific to the particular body part or organ (Norton et al. 2011; Rabey et al. 2015). The whole‐body RFR for mice is 40–60 Hz, 85–92 Hz for the abdomen, 237–253 Hz for the head, and 711–727 Hz for the thorax. Because jackhammer vibrations transmit farthest through buildings at frequencies below 6000 Hz (Norton et al. 2011), the mouse RFR values support a susceptibility to trauma induced by construction vibration. These vibrations within the RFR are likely to elicit a hypothalamic–pituitary–adrenal (HPA) stress response.

Stress activates neuroendocrine responses via the HPA axis that elevate corticosteroids and catecholamines (Schmidt et al. 2003). Corticosterone increases can lead to implantation failure and norepinephrine reduces uterine and placental blood flow. Stress experienced during pregnancy also can be transmitted epigenetically (Babenko et al. 2015) and thereby potentially prolong the response period resulting from any specific incident. A hyporesponsive period extends from birth to P12 for mouse pups, in which plasma corticosterone is not elevated in response to stress (Schmidt et al. 2003), but maternal deprivation for pups can overcome this insensitivity and lead to elevated plasma corticosterone (Enthoven et al. 2010; Daskalakis et al. 2014). This hyporesponsive period also occurs together with decreased expression of the STREX variant of the BK channel in anterior pituitary cells and adrenal chromaffin cells (Lai and McCobb 2006). A further indication of BK channel involvement can be seen in the blunted action from the HPA axis during restraint stress for BK^KO^ mice (Brunton et al. 2007). An attenuated HPA responsiveness may disadvantage BK^KO^ pups during maternal stress associated with construction events, especially if the WT and het pups do respond (Fig. 3). The disrupted circadian rhythms (Meredith et al. 2006) and hyporesponsive HPA axis (Brunton et al. 2007) of BK^KO^ mice may be linked through the circadian regulation of the HPA axis and metabolic control, perhaps similar to the circadian and HPA‐related control of insulin release (Dickmeis et al. 2013; Perelis et al. 2015; Tsang et al. 2016).

### Development in the absence of BK

As a K^+^ channel, the BK protein acts to make the cell membrane electrical potential (*V*_m_) more negative on the inside compared with the outside, thereby altering the electrochemical driving force for ion flow through ion channels and also influencing channels activated by changes in *V*_m_. BK channel opening decreases the opening of channels activated by *V*_m_ depolarization including classes of channels (Yu et al. 2005) for Na^+^ (Na_V_1) and Ca^2+^ (Ca_V_1, Ca_V_2, Ca_V_3), as well as K^+^ (K_Ca_1.1{BK}, K_V_1, K_V_2, K_V_3, K_V_4, K_V_7, K_V_10, K_V_11, K_V_12). Through this *V*_m_ modulation of channel opening and closing kinetics, the BK channel can either slow action potential frequency by prolonging the after hyperpolarization or increase this frequency by shortening action potential duration (Jaffe et al. 2011; Montgomery and Meredith 2012; Montgomery et al. 2013; Duncan et al. 2015). Opening Ca_V_ channels leads to increased cell Ca^2+^ that initiates contraction of smooth muscle cells and release of vesicularly stored hormones from endocrine cells (González et al. 2012; Contreras et al. 2013; Petkov 2014). BK and Ca_V_ channels have an intimate feedback regulation in which opening Ca_V_ activates BK by depolarizing *V*_m_ and increasing cell Ca^2+^. Opening these BK channels hyperpolarizes *V*_m_ leading to closing of Ca_V_ and BK. Adding to the intricacy of this regulatory coupling, the more negative *V*_m_ resulting from opening of BK increases the driving force for conductive Ca^2+^ entry, thereby prolonging the rise in cell Ca^2+^. This increased Ca^2+^ not only allows the signaling action of Ca^2+^ to persist, but also maintains BK channel opening in the face of hyperpolarization. Loss of the BK channel limits the range of response for these cells, which may result in developmental bottlenecks that threaten survival and growth. The unplanned stress that occurred during construction (Fig. 3) presumably combined with the absence of BK to generate several threats to developmental progression.

Development occurs over an extended time from conception to adult, consisting of three broad periods: prenatal, postnatal, and after weaning. Within the prenatal period, development progresses from fertilization of the egg by a sperm, to implantation of the blastocyst in the endometrium, through generation of the placenta, and fetal growth. Postnatal development exhibits a series of milestones that progressively allows the pup to gain independence from the dam. Growth after weaning culminates in a full‐sized adult, governed by the metabolic status of that mouse strain and any overt gene manipulation.

### Influences of BK during the prenatal period

The lack of the BK channel drastically reduces male fertility, such that only 1 in 20 BK null male mice sired a litter with wild‐type female mice (Meredith et al. 2004). The cause of this male infertility likely stems from erectile dysfunction (Werner et al. 2005); but concurrently, sperm maturation (capacitation) occurs progressively along the female reproductive tract (Pritchett and Taft 2007; Aitken and Nixon 2013) with flagellar motility modulated by K^+^ channels (Lishko et al. 2012). BK appears to play a role in human sperm motility, but a K^+^ channel with similar amino acid sequence to BK, K_Ca_5.1 (*Kcnu1*), instead is essential for male fertility in the mouse (Miller et al. 2015). Because sperm differentiation occurs using the diploid genome of the spermatid rather than the ultimate haploid genome of the individual sperm (Pritchett and Taft 2007), (+/−)*Kcnma1* mouse sperm membranes should contain functional BK channels such that dysfunction of sperm motility should not contribute substantively to the observed non‐Mendelian distribution of genotypes for pups born (Table 3B, Fig. 5). If gene dosage contributes to producing a sufficient number of BK channels, then all of the sperm would be hypomotile to the same extent.

Implantation of a blastocyst into the endometrium is the initial step leading to placental development (Wang and Dey 2006; Pritchett and Taft 2007; Hui 2012; Bolon and Ward 2015). The endometrium modifies the uterine lumen volume by absorbing and secreting fluid using various ion channels including BK (Ruan et al. 2014). Endometrium increases expression of BK (both mRNA and protein) during the midsecretory phase (luteal) of the menstrual cycle (Zhang et al. 2012). Knockdown of BK in the endometrium by siRNA reduces blastocyst attachment and subsequent implantation in mouse uterus (Zhang et al. 2012). Although BK appears to facilitate implantation, the event of implantation leads to an ~twofold decrease in BK mRNA expression for bovine endometrium (Forde et al. 2011). A gene dosage influence in the endometrium seemingly would disadvantage all implantations, but the ability of fhet mice to produce large litters (Fig. 1A) supports the presence of full endometrial function concerning implantation.

Development of the placenta from the trophectoderm of the embryo involves imprinting of some genes such that the maternally inherited allele is used (Wagschal and Feil 2006; Peters 2014; Varmuza and Miri 2015). Analysis of first trimester placenta indicates imprinting of the paternal BK allele (Oudejans et al. 2004), and that the BK gene occurs within a chromosome region gene cluster containing a gene (*STOX1*) associated with preeclampsia (van Dijk et al. 2005). Such imprinting in a (+/−)*Kcnma1* embryo could lead to loss of BK function when the maternal allele is null, but often paternal imprinting in the placenta does not show complete repression (Wagschal and Feil 2006). Stress‐induced alterations of imprinting might be a part of the mechanism that selectively disadvantages het mice (Table 3B, Fig. 5).

The placenta is an intimate collaboration between fetal and maternal tissue that brings the blood circulation of each into close proximity, but still separated (Hui 2012; Bolon and Ward 2015). Control of blood flow into the zone for exchange requires precise regulation that is compounded in the case of multiple fetuses within the uterus, as occurs for mice. Each uterine horn has adequate space for 10 or more implantations (Pritchett and Taft 2007), but adequacy of blood supply could limit viability for some of these sites. Maternal perfusion of the placental sites in the uterus occurs via two distinct arteries in each horn, the uterine branch of the ovarian artery, and the uterine artery (Raz et al. 2012). Fetuses adjacent to other fetuses with spontaneous pathologies exhibit lower maternal blood volume in their placenta indicating a positional disadvantage for obtaining sufficient blood perfusion. In contrast, fetuses adjacent to hypoperfusion mutant fetuses exhibited increased maternal blood volume in their placenta indicating an advantage for growth. Increases in blood flow to the placenta occur through opening of BK channels that dilate uterine smooth muscle cells (Rosenfeld et al. 2009; Rosenfeld and Roy 2012), and oxidative stress suppresses this BK activation (Hu et al. 2016). Any gene dosage effects from the (+/−)*Kcnma1* dams likely would influence blood flow to all of the fetuses equally. The fetus could alter dilation of the arteries perfusing the placenta by releasing signaling molecules, possibly via the giant cell lineage that takes up a position near the maternal spiral arteries. The absence of BK, either for (−/−)*Kcnma1* or by imprinting of (+/−)*Kcnma1*, may remove an inhibitory influence on Ca^2+^‐dependent release mechanisms such that signaling would be more persistent and sensitive to stimulation.

The non‐Mendelian distribution of births (Table 3B, Fig. 5) does not allow a determination of which developmental stage presents the disadvantage to particular pups; that definition will require a careful examination of fetuses at specified points of gestation together with measurement of placental blood perfusion (Papaioannou and Behringer 2012; Raz et al. 2012; Bolon and Carreira 2015). Attention also would need to be given to the position of female fetuses relative to male fetuses because majority male litters exhibited the largest disadvantage (Fig. 5A and B). The shift in disadvantage from fKO pups to fhet pups resulting from the apparent construction stress may be most likely to occur during a later gestational time when multiple factors might interact. Interplay between testosterone signaling (Ryan and Vandenbergh 2002) and loss of the BK influences on signaling that act on placental perfusion might account for the preferential disadvantage within female pup categories. The curious interaction between fhet and fKO mice during construction (Fig. 5C) suggested that other stress‐related factors emanating from fetuses must contribute to intrauterine survival.

### Influences of BK during the postnatal period

The postnatal period exhibits numerous developmental milestones for pups as they acquire greater strength and sensory abilities during the progression toward weaning (Fox 1965; Branchi et al. 2003; Heyser 2004; Turgeon and Meloche 2009). Some of these developmental events likely contribute to the two episodes of pup death (Fig. 2A), presumably occurring due to the lack of BK channels that interferes with a key transition. Postnatal survival demands an ability of the pup to grasp the dam (Turgeon and Meloche 2009) such that the weaker grip strength of BK^KO^ mice (Meredith et al. 2004; Typlt et al. 2013) might limit success at nursing. This weakness could result from impaired signal transmission at the neuromuscular junction where BK channels contribute to modulating Ca^2+^ entry by adjusting both Ca^2+^ channel opening and the driving force for Ca^2+^ entry (Pattillo et al. 2001; Berkefeld et al. 2010; Ford and Davis 2014; Lee et al. 2014). Development of the neuromuscular junction occurs with a structural expansion from P0 to P5 and final maturation at P14 (Sanes and Lichtman 1999). The BK channel influences this progression of neuromuscular junction development apparently via formation of macromolecular complexes with structural components and Ca^2+^ channels (Berkefeld et al. 2010; Lee and Wu 2010; Jepson et al. 2014).

Several sensory abilities expand along with muscular strength in the later postnatal period (Fox 1965; Smith et al. 2002; Branchi et al. 2003; Heyser 2004; Castelhano‐Carlos et al. 2010). Opening of the eyes and the auditory canals allow the pups to see and hear for the first time around P12 to P15. Postural strength indicated by bar holding also beginning around P11 increases and reaches near adult levels at P16 (Heyser et al. 1995). Functional expression of BK channels for inner hair cells of the cochlea commences around P12 at the onset of hearing (Marcotti 2012; Corns et al. 2014). In contrast, vestibular hair cells exhibit BK channels prior to birth (Schweizer et al. 2009), but in some hair cells, BK expression may peak between P6 and P23. The susceptibility of BK^KO^ pups to death in the late nursing periods around P14 (Fig. 2) highlights a developmental period when sensory and motor function nears adult level abilities. Matching sensory input to motor output could be the stress that disadvantages some pups. Successful passage of BK^KO^ pups through this period may result from compensation through activating other K^+^ channels such that survival is not threatened again in the typical vivarium housing environment.

### Influences of BK after weaning

Pup growth after weaning leads to adult size and reproductive capacity. Compared with C57BL/6J mice from commercial sources, the mWT mice in the WSU colony were smaller by ~3 g, whereas the fWT mice were near average in weight (Fig. 6). This selective weight deficit for the mWT mice suggests an environmental influence separate from nutritional sufficiency. The divergence in colony weights by 12 weeks of age supports the presence of three distinct weight classes, a cluster at the mean along with colonies >1 SD larger than the mean and others >1 SD smaller (Fig. 6B & C). With these weight groupings as a guide, the WSU colony mWT mice may have a lower relative propensity to develop obesity as might two of the Harlan mouse colonies (C57BL/6J*OlaHsd*, Jerusalem, Israel and C57BL/6N*Hsd*, Horst, the Netherlands).

The BK^KO^ mice in the WSU colony uniformly grew slower and attained a smaller adult weight than their WT littermates (Figs. 6, 7, 8, 9, 10). The 5% shorter tibia length of C57BL/6N BK^KO^ male mice (Illison et al. 2016) indicates that this lower weight occurred as an overall smaller mouse, both weight and skeletal frame. Presumably this reduced body size resulted as a consequence of lacking BK or from an adaptation to maintain function in the face of this absence. In either case, these BK^KO^ mice also adopted a distinct body composition with higher fat proportion (Table 4, Fig. 12). The many cell types using BK channels to control cellular processes (González et al. 2012; Contreras et al. 2013) must adapt in BK^KO^ mice to accomplish maintenance of an overall body composition, though possibly in a suboptimal manner.

Regulation of body weight involves numerous interacting signals from many cells types (Ramsey et al. 2007; Schneeberger et al. 2014; Tsang et al. 2014; Perelis et al. 2015; Rutkowski et al. 2015). Disturbances of circadian rhythm emanating from the superchiasmatic nucleus together with suppression of the HPA axis in BK^KO^ mice (Meredith et al. 2006; Brunton et al. 2007) likely contribute to the lower weights attained by these mice (Fig. 9; Illison et al. 2016; Sausbier et al. 2004). Other factors also must contribute, because BK^KO^ mice in a FVB/NJ background attain weights similar to wild‐type littermates after 5 weeks of age (Meredith et al. 2004), while BK^KO^ mice on a mixed SV129/C57BL6 or C57BL/6N background were smaller than wild type (Typlt et al. 2013; Illison et al. 2016) similar to the WSU colony (Fig. 6). The earlier and more frequent pauses in weight gain for BK^KO^ mice (Fig. 7) suggested occurrences of stronger appetite suppression in the absence of the BK channel. The appearance of weight subgroups as growth slows with age (Fig. 10) further suggested that variations in set point control for weight occur on the scale of weeks. The rate of attaining the set‐point weight likely includes BK channel control of hormone and neurotransmitter release.

Insulin occupies a central position in regulation of metabolism with a role in the development and persistence of obesity (Kahn et al. 2006; Czech et al. 2013). Involvement of BK channels in insulin release (Braun et al. 2008; Houamed et al. 2010; Düfer et al. 2011; Félix‐Martínez and Godínez‐Fernández 2014) may act to alter body weight and fat proportion (Czech et al. 2013). The association of *Kcnma1* with human obesity likely depends in part on the presence of BK channels in adipocytes (Hu et al. 2009; Jiao et al. 2011; Illison et al. 2016). Fasting blood glucose concentration was similar for WT and BK^KO^ mice (Fig. 11; Dufer et al. 2011), but in the mixed SV129/C57BL6 background, WT fasting blood glucose is higher (~7.5 mmol/L; Dufer et al. 2011) than that in the WT C57BL/6J mice from this study (~4.5 mmol/L, Fig. 11) and WT mice in a C57BL/6N background (~5.5 mmol/L; Illison et al. 2016). Response to ipGTT for WSU colony C57BL/6J mice supported an enhanced handling of glucose by BK^KO^ mice (Fig. 11) suggesting a higher or more persistent insulin release during the first and/or second phase of the response to glucose. In contrast, BK^KO^ mice in a mixed SV129/C57BL6 background show a weakened handling of glucose, although blood glucose returns to WT levels after 2 h (Düfer et al. 2011). Isolated pancreatic islets from these BK^KO^ SV129/C57BL6 mice release less insulin in response to glucose compared to WT (Düfer et al. 2011), whereas inhibition of BK channels increases insulin release in Swiss Weber mouse islets (Houamed et al. 2010) and either decreases or increases insulin release in human islets depending on the opening of other K^+^ channels in the *β* cells (Braun et al. 2008). Mice with an adipocyte‐specific knockout of BK exhibit identical ipGTT responses compared with WT (Illison et al. 2016), supporting the lack of involvement of adipocyte BK channels in glucose challenges. Observations of less insulin release during acute BK inhibition (Braun et al. 2008) or in the absence of the BK protein (Düfer et al. 2011) as well as more insulin release with BK inhibition (Houamed et al. 2010) support an intricate interplay between BK channels and other K^+^ channel types in modulating the activation and magnitude of Ca^2+^ influx that leads to *β*‐cell insulin release (Félix‐Martínez and Godínez‐Fernández 2014).

The impaired handling of glucose by WT C57BL/6J mice (Toye et al. 2005; Berglund et al. 2008; Fergusson et al. 2014; Fisher‐Wellman et al. 2015) illustrates the importance of the metabolic background for interpreting experimental results (Schneeberger et al. 2014; Fontaine and Davis 2016; Yeadon [Link]), especially with knockout mice. Despite the spontaneous truncation mutation to a key mitochondrial enzyme, nicotinamide nucleotide transhydrogenase (*Nnt*), C57BL/6J mice apparently compensate for this deficiency such that reduction of NADP^+^ to NADPH remains typical (Freeman et al. 2006; Wong et al. 2010; Ronchi et al. 2013). The ability of the WSU colony WT C57BL/6J mice to handle the initial rise in glucose may be impaired as indicated by the relatively large increase of blood glucose (peaking at ~20 mmol/L during ipGTT), but it decreased to control levels within 2 h suggesting an overall conserved tolerance to glucose (Fig. 11). The age of these mice (6–7 weeks) may be a distinguishing feature, because a study using C57BL/6J and C57BL/6N mice at 8 weeks of age indicates similar glucose tolerance even though C57BL/6N mice have full length *Nnt* (Wong et al. 2010). Several of the studies indicating impaired glucose tolerance for C57BL/6J mice used mice 12 weeks of age or older (Toye et al. 2005; Berglund et al. 2008; Fergusson et al. 2014; Fisher‐Wellman et al. 2015). Together, developmental age and background strain genetics likely contribute to the robustness of glucose handling.

Alterations in fat storage depend on insulin signaling (Czech et al. 2013; Rutkowski et al. 2015) such that changes in body composition provide indications of dysregulation. Induction of obesity by feeding a high‐fat diet was dramatically blunted in C57BL/6N BK^KO^ male mice, which was partially replicated by silencing BK channel expression only in adipocytes (Illison et al. 2016). This protective influence of losing BK activity may result in part from suppression of preadipocyte proliferation (Hu et al. 2009). Weight gain for the wild‐type male littermates fed with the control diet (Illison et al. 2016) was similar to mice in the lower weight class (Fig. 6B). Measurements of body composition by QMR (Fisher‐Wellman et al. 2015) indicate a higher fat proportion for C57BL/6J mice (~0.145) than for C57BL/6N mice (~0.085). Similar QMR measurements for WSU colony C57BL/6J mice (Table 3, Fig. 12) indicated a fat proportion of 0.074 for males and 0.097 for females. These comparatively lower fat proportions for C57BL/6J mice suggested the likelihood of contributions to fat storage from additional factors such as housing conditions. A whole body extraction method for determining fat composition indicates a fat proportion of ~0.26 for male C57BL/6N mice with male BK^KO^ mice having a lower fat proportion of ~0.12 (Illison et al. 2016). In this study, QMR measurement indicated that both male and female C57BL/6J BK^KO^ mice exhibited higher fat proportion (~0.02 larger) compared to WT (Table 3, Fig. 12). The ability of the QMR method to provide in vivo measures of body composition may allow detection of subtle changes that presage susceptibilities for pathologic changes encountered with metabolic stress. Together, the lower relative weight of WSU colony mWT mice and this specific body composition may define a distinct metabolic subtype that governs weight gain.

The opposing outcomes reported for glucose handling and body composition may relate to the signaling set point determined by the contributing neural and endocrine cells. In particular, BK channel modulation of insulin release from pancreatic *β*‐cells could lead to higher or lower plasma insulin concentrations. For those signaling contexts in which loss of BK channel activity (channel blocker or BK^KO^) led to increased insulin release (Braun et al. 2008; Houamed et al. 2010; Fig. 11), the primary cellular role of these BK channels would have been to lower insulin release likely via limiting voltage‐gated Ca^2+^ channel opening by hyperpolarizing *V*_m_ using a relatively large activation of BK. In contrast, loss of BK channel activity that leads to smaller insulin release (Braun et al. 2008; Düfer et al. 2011) likely would occur in situations where BK channels act to increase the electrochemical gradient for Ca^2+^ entry via a small and precise activation of BK that prolongs a rise in intracellular Ca^2+^ activity with a consequent higher overall insulin release. The possibility that BK channel activation can lead to either increased or decreased insulin release depending on the signaling context of the *β* cell makes BK a pivotal protein for controlling plasma insulin levels.

## Conclusions

Overall, the lack of BK channel activity influences many cellular systems by removing one means to adjust *V*_m_, often resulting in altered signaling that disrupts optimal function. Prenatally, survival was impaired primarily for female mice with an influence from construction‐related stress that shifted the burden from fKO to fhet mice. Postnatally, a deficit in grip may have limited the ability of pups to obtain adequate nutrition. Long‐term health may have been compromised by derangements in the release of insulin (and possibly other neuroendocrine signaling molecules) leading to signaling that diverts energy supplies from growth to fat storage. Whether these specific growth outcomes are observed when BK channel activity is absent likely depends on the genetic background as well as vivarium‐related environmental factors that provide a context for the cellular signaling that initiates physiological responses. Finally, monitoring reproductive success and weight gain can provide key information to the analysis of any transgenic or knockout animal model.

## Conflict of Interest

None declared.
